# An automated pipeline to generate initial estimates for population Pharmacokinetic base models

**DOI:** 10.1007/s10928-025-10000-z

**Published:** 2025-11-06

**Authors:** Zhonghui Huang, Matthew Fidler, Minshi Lan, Iek Leng Cheng, Frank Kloprogge, Joseph F Standing

**Affiliations:** 1 Great Ormond Street Institute of Child Health, University College London, London, UK; 2 Novartis Pharmaceuticals Corporation, Fort Worth, Texas USA; 3 Institute for Global Health, University College London, London, UK; 4 Great Ormond Street Hospital for Children, London, UK

**Keywords:** Initial estimates, Population pharmacokinetics, Automated modeling, Sparse data

## Abstract

Nonlinear mixed-effects models rely on adequate initial parameter estimates for efficient parameter optimization. Poor initial estimates can result in failed model convergence or termination with incorrect parameter estimates. Non-compartmental analysis (NCA) and other manual methods have typically been used to derive initial estimates for pharmacokinetic (PK) parameters. However, NCA struggles with sparse data and recent advances in automated modeling increasingly emphasize the need for initial estimates that require no user input. This study aimed to develop an integrated pipeline for the computation of initial estimates applicable to various data types and model structures. The designed pipeline incorporated a custom-designed algorithm that leveraged data-driven methods to generate initial estimates for both structural and statistical parameters in population pharmacokinetic (PopPK) base models. The pipeline’s performance was evaluated across twenty-one simulated datasets and thirteen real-life datasets. The results suggested that this pipeline performed well in all test cases. Initial estimates recommended by the pipeline resulted in final parameter estimates closely aligned with pre-set true values in simulated datasets or with literature references in the case of real-life data. This study provides an efficient and reliable tool for delivering PK initial estimates for population pharmacokinetic modeling in both rich and sparse data scenarios. An open-source R package has been created.

**Supplementary Information:**

The online version contains supplementary material available at 10.1007/s10928-025-10000-z.

## Introduction

Population pharmacokinetic (PopPK) model analysis involves constructing mathematical and statistical models and performing parameter estimation to characterize the absorption, distribution, metabolism, and elimination of drugs. It is necessary to provide initial estimates to the parameter optimizers, which will then undergo iterative parameter optimization and estimation. Initial estimates are usually determined by the modeler. A common approach is to conduct a preliminary exploration of data from one or more individuals or to set the initial estimates based on published literature [1]. However, modeler-led approaches lack automation, rendering them time-consuming and difficult to standardize.

Some PopPK modeling tools offer features to automatically set initial estimates. For example, Monolix optimizes initial estimates through a custom optimization on pooled data disregarding inter-individual variability (IIV) [2]. This process needs to collect initial values from the panel as starting points of optimization. Babelmixr2 [3], a package that can connect nlmixr2 with PKNCA [4], computes initial estimates by performing non-compartmental analysis (NCA) and applying empirical settings. Nevertheless, it may be sensitive to the types of data used, particularly for sparse data [5]. NONMEM lacks a built-in automatic setting for initial estimates, but external tools like pyDarwin can be utilized. As an automated PopPK modeling tool, pyDarwin can incorporate initial estimates along with other model features into the search space for optimization within an evolutionary algorithm [6]. Another automatic tool, Pharmpy, requires users to input initial values for the starting model [7], and in one practice, NCA’s results were used as a reference for the starting models’ initial estimates [8].

Other approaches based on data exploration are available. The single-point method, an earlier approach that utilizes a specific time point to predict trough concentrations [9, 10], along with more recent practices that estimate AUC using a single trough concentration [11, 12], was a potential solution for handling sparse data. Performing NCA on pooled data is another choice. The practice involves treating all data as if from a single subject [13] and may involve combining data points at the same time interval [14]. The graphic methods [15, 16] offer a flexible approach applicable to both sparse and rich data. For complex models, especially those where multiple parameters lack pre-determined values, parameter sweeping can be useful. It tests a user-defined range of possible parameter values and evaluates their outputs to select a suitable value with the best performance [17, 18].

There is a clear gap in tools that can automatically generate initial estimates without user input, which are universal, time-efficient, and effective for both manual modeling and automated modeling algorithms. Hence, the pipeline presented here aimed to provide references for initial estimates of base model parameters when no prior information from other sources is available and accommodate a wide range of PK scenarios, including those involving sparse data. This was accomplished through data exploration-based parameter analysis, including adaptive single-point method, graphic methods, naïve pooled NCA, and parameter sweeping.

## Methods

### Pipeline overview

**Fig. 1:**
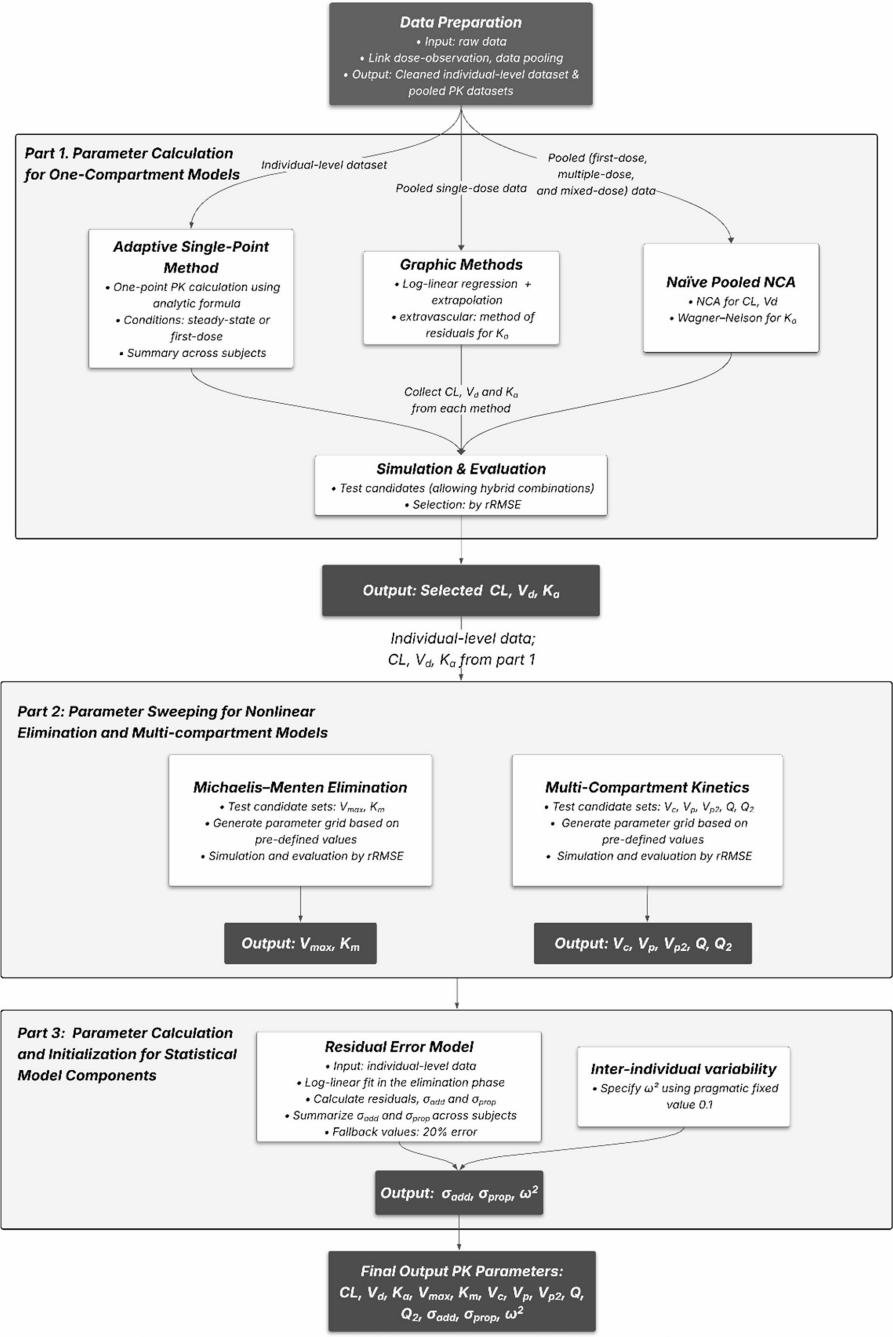
Workflow diagram of the automated pipeline for generating initial estimates of commonly used PK parameters. The workflow consists of three main parts: the first focuses on computing one-compartment parameters, including clearance (CL), volume of distribution (V_d_), and absorption rate constant (K_a_) (the top panel); the second part concentrates on extended structural parameters of multi-compartment and Michaelis-Menton elimination models (the middle panel). These include: V_c_ (central volume of distribution), V_p_ (volume of distribution of peripheral compartment), Q (inter-compartmental clearance), V_p2_ (volume of distribution of the second peripheral compartment), Q_2_ (the second inter-compartmental clearance), maximum elimination rate (V_max_), and Michaelis constant (K_m_); The final part (the bottom panel) handles statistical model components, including σ_add_ (standard deviation of additive residual error model), σ_prop_ (standard deviation of proportional residual error model), and ω^2^ (variance of IIV). The rRMSE refers to the relative root mean square error

A pipeline was established to compute PK parameters from datasets formatted according to nlmixr2 data standards (see Fig. 1). It comprised three main parts: (1) parameter calculation for one-compartment models, (2) parameter sweeping for nonlinear elimination and multi-compartment models, and (3) parameter calculation and initialization for statistical model components.

*Part 1* analyzed base parameters (clearance (CL), volume of distribution (V_d_), and absorption rate constant (K_a_)) through three main approaches:

1. **Adaptive single-point method**: This approach was originally inspired by calculating parameters from a single concentration point [9, 10]. This study redesigned the single-point approach to incorporate data points under both initial-dose and steady-state conditions. An “extended phase” was added to address parameters not calculated in the base phase, providing the pipeline with the flexibility to handle different data types.
2. **NCA**: This approach incorporated the Wagner-Nelson method [19] into the NCA framework to assist in calculating the K_a_ to derive the necessary pharmacokinetic parameters.
3. **Graphic methods**: These methods were built upon established methodologies for one-compartment models [15, 16].

*Part 2* focused on model-specific parameters in more complex models, using a parameter sweeping approach. A range of candidate values was tested by simulating model-predicted concentrations, and those with the best predictive performance measured as relative root mean squared error (rRMSE) were selected. In *Part 3*, a data-driven approach was employed to calculate residual unexplained variability (RUV) when sufficient data were available, with fallback to pragmatic defaults otherwise. For IIV, pragmatic default values were used to facilitate model initialization. Details of each workflow are also provided in Supplementary Material 1.

## Pipeline development: data preparation

The pipeline began by processing observation records to assign dosing information, identify administration routes (bolus, infusion or extravascular), and calculate time after the last dose (TAD**)** (Supplementary Fig. 1, Material 1). The resulting data, hereafter referred to as individual-level data, served as the foundation for adaptive single-dose methods, parameter sweeping and residual error estimation.

A naïve pooling approach was then applied to process concentration-time data for subsequent estimation of elimination half-life (hereafter “half-life”) and analyses using NCA and graphic methods. Pooling was based on three groups: first-dose data, non-first-dose data (considered to be multiple-dose data), and mixed-dose data that included both types of dosing occasions. All concentration-time data within each group were binned and pooled based on TAD, using predefined time windows with a default number of 10, considering that adequate PK analysis typically requires 3–4 points after peak time (T_max_), and 2 points before T_max_ for extravascular formulations [20, 21]. These intervals were generated by dividing unique time points into quantiles, with each group containing an approximately equal number of time points. If fewer than ten unique time points were available, the intervals were adjusted to match the actual number. Within each time window, the median time and drug concentration were calculated for each group, serving as representative values for time and concentration within that time window.

### Pipeline development Part 1: parameter calculation for one-compartment models

1. ***Adaptive single-point method.*** The adaptive single-point method was designed as a framework for calculating PK parameters from single-point samples per individual, followed by population-level summarization. The framework was divided into two phases: the base phase, which computed parameters including CL and V_d_, and the extended phase, which addressed cases where CL or V_d_ could not be calculated due to limited data and also included estimation of the absorption rate constant (K_a_), which was not covered in the base phase. The overall workflow of this framework is shown in Supplementary Fig. 2 (Material 1).

- ***1.1 Base phase.*** Post-first-dose and steady-state data were extracted from individuals. Steady state was defined as being achieved following administration of regularly spaced doses covering at least five half-lives or five doses, with dose intervals and fluctuations within ± 25% of the median. Half-life was estimated through linear regression on naïve pooled data. V_d_ was calculated as the ratio of the dose to the concentration observed at the first sampling point after the initial dose. This point was required to be collected within 20% of the half-life after dosing, during which concentration drops approximately 13% under linear elimination, to approximate the time-zero concentration. Maximum (C_ss, max_) and minimum (C_ss, min_) concentrations were extracted from the same interval under steady state, and their mean (C_ss, avg_) was used to calculate CL (see Table 1). CL was subsequently derived solely based on the C_ss, max_ or C_ss, min_, and this calculation was only applicable to intravenous cases. A geometric mean with a trim value of 0.05 (i.e., removing the top and bottom 2.5% of the data) was used to summarize PK parameters derived from individuals, given as a more robust alternative, resistant to outliers approach [22].

- ***1.2 Extended phase.*** Missing CL or V_d_ in the base phase were derived using the estimated half-life in the extended phase (see Table 1). When both were missing, the central volume of distribution (V_c_) was used as a substitute for V_d_. V_c_ was estimated using a C_max_-based approach, calculated as the ratio of dose to the C_max_ within 20% of the half-life following a single dose. For multiple doses, the accumulation ratio (R_ac_) was applied to adjust C_ss, max_ back to C_max_. K_a_ was calculated by solving the analytical concentration-time equations for a one-compartment pharmacokinetic model after a single or multiple doses. Concentration data from the absorption phase (individual sampling points at sampling times ≤ peak time) were used. CL and V_d_ in the equations were obtained from previous steps, and bioavailability was assumed to be 1. K_a_ was subsequently determined within a wide range of values (0-1000) using Brent’s method implemented in R’s uniroot function [23]. K_a_ and V_c_ were summarized by calculating the trimmed geometric mean of individual values.

**Table 1:** Available methods for pipeline one-compartment pharmacokinetic calculations

Method	Calculation Description	Equations
Adaptive single-point method (base phase)	• V_d_ is calculated using C_1_ after administration, provided it occurs within 0.2 times the estimated half-life (approximately 13% elimination). This calculation is only applicable to intravenous cases. • CL is calculated based on the mean of C_ss, max_ and C_ss, min_. A single point of C_ss, max_ and C_ss, min_ can be used for CL calculation in intravenous cases. τ corresponds to the most recent dosing interval.	$\:{V}_{d}=\frac{\text{Dose}}{{C}_{1}}$ $\:CL=\frac{\text{Dose}}{{C}_{ss,avg}\times\:\tau\:}$ $\:{\text{C}}_{ss,min}={C}_{ss,max}\times\:{e}^{-\frac{ln\left(2\right)}{{t}_{1/2}}\tau\:}\left(\text{b}\text{o}\text{l}\text{u}\text{s}\right)$ $\:{C}_{ss,min}={C}_{ss,max}\times\:{e}^{-\left[\frac{\text{ln}\left(2\right)}{{t}_{1/2}}\right]\cdot\:\left({\uptau\:}-{t}_{\text{inf}}\right)}\left(infusion\right)$ $\:{C}_{ss,avg}=\frac{{C}_{ss,max}+{C}_{ss,min}}{2}$
Adaptive single-point method (extended phase)	• If V_d_ and CL cannot be determined from the base part, then estimated half-life is introduced. • V_c_ is estimated using observed C_max_ values, with R_ac_ applied to convert the C_max, ss_ to C_max_. • For extravascular cases, K_a_ is determined by solving one-compartment equations using observed concentrations during the absorption phase, with F_bio_ assumed to be 1.	$\:{V}_{d}=\frac{CL\cdot\:{t}_{1/2}}{\text{l}\text{n}\left(2\right)}$ $\:{V}_{c}=\frac{Dose}{{C}_{max}}$ $\:{C}_{max}=\frac{{C}_{max,ss}}{{R}_{ac}}\left(R_{ac} = \frac{1}{1 - e^{-k_e \tau}}\right)$ $\:{C}_{t}=\frac{{F}_{bio}Dose{k}_{a}}{{V}_{d}\left({k}_{a}-{k}_{e}\right)}({e}^{-{k}_{e}t}-{e}^{-{k}_{a}t})\:$ $\:{C}_{t}=\frac{{F}_{bio}Dose{k}_{a}}{{V}_{d}\left({k}_{a}-{k}_{e}\right)}(\frac{{e}^{-{k}_{e}t}}{1-{e}^{-{k}_{e}\tau\:}}-\frac{{e}^{-{k}_{a}t}}{1-{e}^{-{k}_{a}\tau\:}})$
NCA	• For single-dose data, AUC_0-∞_ is used for CL calculation. • For data after multiple doses, AUC_0-τ_ is for CL calculation. V_z_ is based on the ratio of CL and λ_z_	$\:AU{C}_{0-{\infty\:}}={\int\:}_{0}^{tlast}{C}_{p}\hspace{0.17em}dt+\:\frac{{C}_{last}}{{\lambda\:}_{z}}$ $\:AU{C}_{0-\tau\:}={\int\:}_{0}^{\tau\:}{C}_{p}\hspace{0.17em}dt$ $\:CL=\frac{\text{1}}{AUC}$ $\:{V}_{z}=CL/{\lambda\:}_{z}$
Graphic methods (IV)	• It is for single-dose analysis. • V_extrap_ is calculated as the inverse of the y-intercept obtained by extrapolating the terminal phase line. CL is derived from the regression of the terminal phase.	$\:{V}_{extrap}=\frac{1}{{Y}_{intercept}}$ $\:CL={\lambda\:}_{z}\:\times\:{V}_{extrap}$
Graphic methods (Extravascular, method of residuals)	• Concentration at the elimination phase is extrapolated, and C_residual_ is calculated extrapolated concentration C_extrap_ minus concentration C_t_ on the profile. The slope of the residual line represents the K_a_. • V_d_ is approximated as Dose/C_extrap_ assuming K_a_ > > K_e_	$\:{C}_{extrap}=\:{\:C}_{0}{e}^{-{k}_{e}t}\:$ $\:({\:C}_{0}=\frac{{F}_{bio}Dose{k}_{a}}{{V}_{d}\left({k}_{a}-{k}_{e}\right)})$ $\:{C}_{residual}={{C}_{extrap}-\:C}_{t}$ $\:{ln(C}_{residual})=\:ln{\:C}_{0}-{k}_{a}t$
Wagner-Nelson method	• Cumulative absorption exposure is calculated. The fraction of absorption is calculated based on all exposure and cumulative absorption exposure at each time t. The magnitude of the slope of the fraction remaining to be absorbed line in the natural logarithm scale is K_a_.	$\:{\text{AUC}}_{0-t}={\sum\:}_{i=1}^{n}\left(\frac{{C}_{i}+{C}_{i-1}}{2}\right){\Delta\:}{t}_{i}$ $\:F\:(\text{t)}=\frac{{C}_{t}+{k}_{e}\cdot\:{\text{AUC}}_{0-t}}{{k}_{e}\cdot\:{\text{AUC}}_{0-{\infty\:}}}$ $\:\text{F'(t)}=1-\:F\left(t\right)$ $\:{k}_{a}=-\text{slope of }(\text{ln}\left(\text{F'(t)}\right)\text{vs}\text{.}\text{}\text{t)}$

CL: clearance, C_ss, avg_: average steady-state concentration, C_ss, max_: maximum steady-state concentration, C_ss, min_: minimum steady-state concentration, τ: dosing interval, t_1/2_: half-life, t_inf_: infusion time, V_d_: volume of distribution, C_0_: initial concentration, C_1_: first concentration point, V_c_: central compartment volume, R_ac_: accumulation ratio, λ_z_: terminal elimination rate constant, C_t_: concentration at time t, F_bio_: bioavailability, k_a_: absorption rate constant, k_e_: elimination rate constant, AUC_0−∞_: area under the curve from time zero to infinity, AUC_0−τ_: area under the curve within a dosing interval, C_p_: plasma concentration, C_last_: the last measurable concentration, V_z_: volume of distribution based on terminal phase, V_extrap_: extrapolated volume of distribution, Y_intercept_: intercept of regression line, C_extrap_: extrapolated concentration, C_residual_: residual concentration.

2. ***Naïve pooled NCA.*** Naïve pooled normalized concentration-by-dose data were used. The area under the curve (AUC) was calculated using the “linear-up log-down” trapezoidal rule. The elimination rate constant (K_e_) was determined by using a best-fit strategy [24] based on log-linear regression of the terminal phase. For single-dose data, AUC from time 0 to infinity (AUC_0-∞_) was used for CL calculation, while for multiple-dose data, AUC_0-τ_ was applied where τ was defined as the most commonly used dosing interval determined by frequency of administration (see Table 1). CL was calculated by dividing the dose (standardized to 1) by the AUC, and the volume of distribution of the terminal phase (V_z_) was calculated using the formula V_z_ = CL/K_e_. For the extravascular case, K_a_ was estimated by the Wagner-Nelson method [19]. The cumulative drug exposure at time AUC_0-t_ was calculated, and a linear regression analysis on the fraction of the drug that remained unabsorbed during the absorption phase was performed to determine the K_a_. A detailed workflow of the naïve pooled NCA process is provided in Supplementary Fig. 3 (Material 1).

3. ***Graphic methods.*** Naïve pooled first-dose data were used for this analysis. The plasma drug concentration versus time data was first plotted on a semi-logarithmic scale. Linear regression was performed on the terminal elimination phase, and the slope was used to estimate K_e_, from which the half-life (t_1/2_) was derived. In the case of intravenous administration, the intercept, extrapolated to the y-axis, was used to calculate V_d_. For extravascular administration, the method of residuals was employed to determine the K_a_. This involved identifying the terminal elimination phase and subtracting it from the total plasma concentration-time curve, leaving the residuals corresponding to the absorption phase. The semi-logarithmic plot of these residuals was then used to calculate K_a_. Detailed equations and workflow are listed in Table 1 and Supplementary Fig. 4 (Material 1) separately.

***Part 1 evaluation and selection.*** Only one set of parameters was selected as final recommendations for one-compartmental parameters based on their predictive performance in a one-compartment linear model. The pipeline also allowed a hybrid combination, where each parameter (i.g., CL, V_d_ and K_a_) could be selected independently from different methods. The predictive performance was examined through rRMSE [25–27], as shown in the following equation:

$$\begin{array}{c}\:rRMSE\:\left[\%\right]\:\:=\:100\:\times\:\\\sqrt{\:\frac1n\:\sum\:_{i=1}^n\left(\frac{\left({predicted\:concentration}_i-{observed\:concentration}_i\right)^2}{\left(\frac{{predicted\:concentration}_i+{observed\:concentration}_i}2\right)^2}\right)}\end{array}$$

Where i refers to the time point, n is the total number of time points.

The set with the lowest rRMSE was selected as pipeline initial estimate recommendations for one-compartment nonlinear mixed-effects modeling and utilized to inform parameter sweeping in *Part 2* of the pipeline.

### Pipeline development Part 2: parameter sweeping for extended pharmacokinetic models

In addition to the basic one-compartment model, this pipeline provided initial estimates for extended pharmacokinetic models, including nonlinear elimination (modeled as Michaelis–Menten kinetics) and multi-compartment structures. During parameter sweeping, it defined parameter ranges, constructed a parameter grid and systematically simulated each combination using individual-level data to identify the best-fitting parameters.

**Fig. 2:**
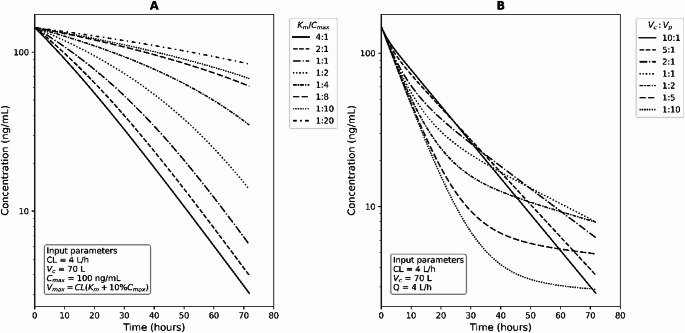
Example simulation outputs from a parameter sweep exploring different K_m_/C_max_ ratios (**A**) and V_c_/V_p_ ratios (**B**). Panel A shows simulation results using K_m_/C_max_ ratios ranging from 4:1 to 1:20, modeled using a one-compartment model with Michaelis-Menten elimination. Panel B presents outputs for V_c_/V_p_ ratios ranging from 10:1 to 1:10, simulated in a two-compartment model. The dose event was set as a single intravenous administration of 100 mg. Input parameters included CL = 4 L/h and V_c_ = 70 L, with C_max_ = 100 ng/mL. In this example, C was set as 10% of C_max_ for V_max_ calculation in Panel A, and Q was set equal to CL in Panel B. Other values were also examined during the actual parameter sweeping, including V_max_ calculated at 5%, 10%, 25%, 50%, and 75% of C_max_, and Q scaled to 0.25-, 0.5-, 1- and 2-fold of CL

***Michaelis–Menten elimination***. This pipeline provided initial estimate recommendations for the maximum elimination rate (V_max_), and Michaelis constant (K_m_), needed for nonlinear elimination modeling through parameter sweeping. This process involved a series of simulations using predefined parameter values based on a one-compartment model with Michaelis-Menten elimination, which generated simulated concentration profiles according to the dose and sampling events from input datasets, as illustrated in Fig. 2A. Parameters for simulation were categorized into test parameters (V_max_ and K_m_) and non-test parameters (V_d_ and K_a_). Non-test parameters (V_d_) were fixed based on values obtained from *Part 1*. The test range for K_m_ was scaled relative to C_max_, covering ratios from 4:1 to 1:20. V_max_ was then calculated based on the Michaelis-Menten kinetic equation:

$$\:CL\:=\:\frac{{V}_{max}}{{K}_{m}\:+\:C}$$

Where concentration (C) was tested at 0.05, 0.1, 0.25, 0.5, and 0.75 times C_max_, and CL values were taken from *Part 1*. Through this battery of simulations, the parameters that provided the best-fit performance, measured by rRMSE, were identified as pipeline output.

***Multi-compartmental kinetics***. A similar parameter sweeping was applied to explore the volumes of distribution (V_c_, V_p_, and V_p2_) and inter-compartmental clearances (Q and Q_2_). The simulated concentration profiles were generated using a two- or three-compartment model with first-order kinetics and predefined parameter values. Among these, K_a_, CL, and V_c_ were considered non-test parameters, with values obtained from the outputs in *Part 1*. There were two candidate values for V_c_: one from V_d_ (calculated through single-point, NCA, or graphic methods) and the other from V_c_ (output from adaptive single-point extended phase).

To construct a parameter grid for parameter sweeping, V_p_ was calculated based on a predefined range of V_c_-to-V_p_ ratios, covering 10:1, 5:1, 2:1, 1:1, 1:2, 1:5, and 1:10. For three-compartment models, V_p2_ was calculated using the same set. Q and Q_2_, were scaled relative to CL, with four candidate values tested: 0.25, 0.5, 1 and 2-fold of CL, where CL was from *Part 1*. Simulations were then conducted for each combination within the full parameter grid once the test spaces were determined. Figure 2B illustrates an example of such a simulation using a two-compartment model, where different V_c_/V_p_ ratios were tested to predict concentration profiles. The most appropriate set of parameters was selected based on the rRMSE.

### Pipeline development Part 3: parameter calculation and initialization for statistical model components

Initial estimates for IIV were specified by assigning a pragmatic fixed value of 0.1 for ω^2^. RUV was characterized by using one of two approaches: (1) a data-driven approach using log-linear regression of terminal-phase data, with residuals computed in the original concentration scale, or (2) a fixed-fraction approach in which an expected observation error was assumed to approximate residual variability. In the data-driven approach, a linear regression was applied to the log-transformed concentration-time data from the terminal elimination phase for each subject. By default, the last three concentration-time points were used to minimize the influence of absorption and distribution. The predicted concentration at time t, denoted as $\:{C}_{pred}$, was obtained by back-transforming the fitted values:

$$\:{C}_{pred}=\text{e}\text{x}\text{p}({\beta\:}_{0}-{K}_{e}\bullet\:t)$$

where $\:{\beta\:}_{0}$ is the intercept and $\:{K}_{e}$ is the elimination rate equal to the negative slope of the regression line.

Residuals were calculated as the difference between the observed concentrations $\:{C}_{obs}$ and predicted concentrations $\:{C}_{pred}$. The additive and proportional residual variances were then computed as follows:

$$\:{\sigma\:}_{add}=\sqrt{Var({C}_{obs}-{C}_{pred})}\:\:\:\:\:\:{\sigma\:}_{prop}=\:\sqrt{Var\left(\frac{{C}_{obs}}{{C}_{pred}}-\:1\right)}$$

where $\:{\sigma\:}_{add}$ represents the estimated standard deviation of additive residual error and $\:{\sigma\:}_{prop}$ refers to the estimated standard deviation of proportional residual error.

After obtaining the individual-subject estimated $\:\sigma\:$, a trimmed mean with a trimming proportion of 0.05 was applied to summarize the results, thereby reducing the influence of outliers. For the fixed-fraction method, when data were insufficient for data-driven estimation, empirical default values were used. According to the NONMEM User Guides [28], initial standard deviations could be set as a fraction (e.g., 20%) of the typical observed value, as shown in the following equation:

$$\:{\sigma\:}_{add}=\:CV\%\:\times\:\stackrel{-}{DV}$$

where $\:\stackrel{-}{DV}$ represents the average observed concentration across the entire dataset, and CV% refers to the expected value for percentage error of the observations. The default setting in the pipeline was 20%.

### Data

A total of 21 simulated and 13 real-life datasets were analyzed. The simulated datasets comprised seven intravenous bolus, seven intravenous infusion, and seven oral cases, each of which included four rich, one semi-sparse, and two sparse designs. The pharmacokinetic models included 12 one-compartment linear, three one-compartment nonlinear (with Michaelis-Menten elimination), three two-compartment linear, and three two-compartment nonlinear models. The 13 real-life datasets included eight intravenous and five oral administration profiles, with five rich and eight sparse sampling designs.

**Simulated data.** All simulated datasets are provided in the supplementary material. 15 out of 21 datasets were obtained directly from the nlmixr2data package [29]. Additionally, three rich one-compartment datasets, *Bolus_1CPT*, *Infusion_1CPT*, and *Oral_1CPT* from nlmixr2data, were extended by generating *semi-sparse*, *sparse1*, and *sparse2* datasets for each, respectively. The semi-sparse dataset was created by dividing the original IDs into three groups, where each group included only two sampling points within a single dosing interval following multiple doses. The sampling points differed among the groups at post-dose 2, 4, 6, 8, 12, and 24 h. *Sparse1* datasets had two or three sampling points available in a different dose interval for all IDs after multiple doses, with sampling time after the last dose at 2 (if oral), 20, and 24 h. *Sparse2* datasets had two or three data points collected at 2 (if oral), 20, and 24 h after the single dose.

**Real-life data.** Real-life data consisted of three datasets, *theo_sd*, *theo_md*, and *pheno_sd* sourced from nlmixr2data [29], as well as ten datasets from nine published articles. Information about these ten datasets is detailed in Supplementary Table 1 (Material 2). The concentration-time curves for the simulated and real-life datasets were provided in Supplementary Figs. 1 and 2 (Material 2).

### Pipeline performance

For the simulated dataset, the pipeline was evaluated by re-estimating the simulated cases using nonlinear mixed-effects modeling, applying the true model that generated the data and the initial estimates proposed by the pipeline. Accuracy was defined as the deviation (%), calculated as the absolute relative difference between the final estimate and the true value used in the simulation.

$$\:Deviations\:\left(\%\right)=\left|\frac{\widehat{\theta\:}\:-\:{\theta\:}_{true}}{{\theta\:}_{true}}\right|\:\times\:100\%$$

where $\:\widehat{\theta\:}$ is the final parameter estimate, and $\:{\theta\:}_{true}$ is the true value of the parameter used for simulation.

A threshold of 20%, as an often-used clinical relevance threshold [28, 30], was applied to evaluate whether the final estimates recovered the true values. For each parameter, the proportion of datasets was calculated in which the absolute relative deviation between the final estimate and the true value did not exceed 20%. As an exploratory analysis, a 30% threshold, also commonly used as a reference for determining clinical relevance in practice [31, 32] was used for evaluation. Overall success rates were computed under both thresholds, defined as the proportion of datasets in which all parameters simultaneously met the respective criterion. The pipeline performance was also compared with the following initial estimate designs for simulated datasets. These strategies were:

1. Setting all initial estimates to 1 before back-transformation (expressed as *inits = 1* in the following description), with parameters defined using log-transformation. For example, the initial estimate of CL was specified to 1, which corresponds to setting the log-transformed CL to 1 in the initial condition function.
2. Setting all estimates to 1 before back-transformation, followed by optimizing the initial estimates using optimization methods available in the R statistical environment [33]: nls (Gauss-Newton method), nlm (Newton-type method), and nlminb (quasi-Newton method), (expressed as *inits = nls*,* inits = nlm*,* inits = nlminb* in the following description) through compartmental analysis without considering IIV.

For real-life clinical data, where the true model structure and parameter values were unknown, parameter estimation was conducted using one- and two-compartment models with IIV on all parameters and a combined residual error model. Model performance using the pipeline and the *inits = 1* strategy was then compared. The evaluation focused on assessing the precision of the final parameter estimates obtained using both strategies, as well as the model’s goodness-of-fit, measured by AIC, and computation time. Stochastic approximation expectation-maximization (SAEM) and first-order conditional estimation with interaction (FOCEI) algorithms were used for test work in simulated and real-life datasets.

### Software

The pipeline was developed in R and is available as an R package, nlmixr2autoinit (version 1.0). The code is available on GitHub (https://github.com/ucl-pharmacometrics/nlmixr2autoinit). The nlmixr2 package was used for model parameter estimation.

## Results

### Pipeline output - CL, V_d_, and K_a_

**Fig. 3:**
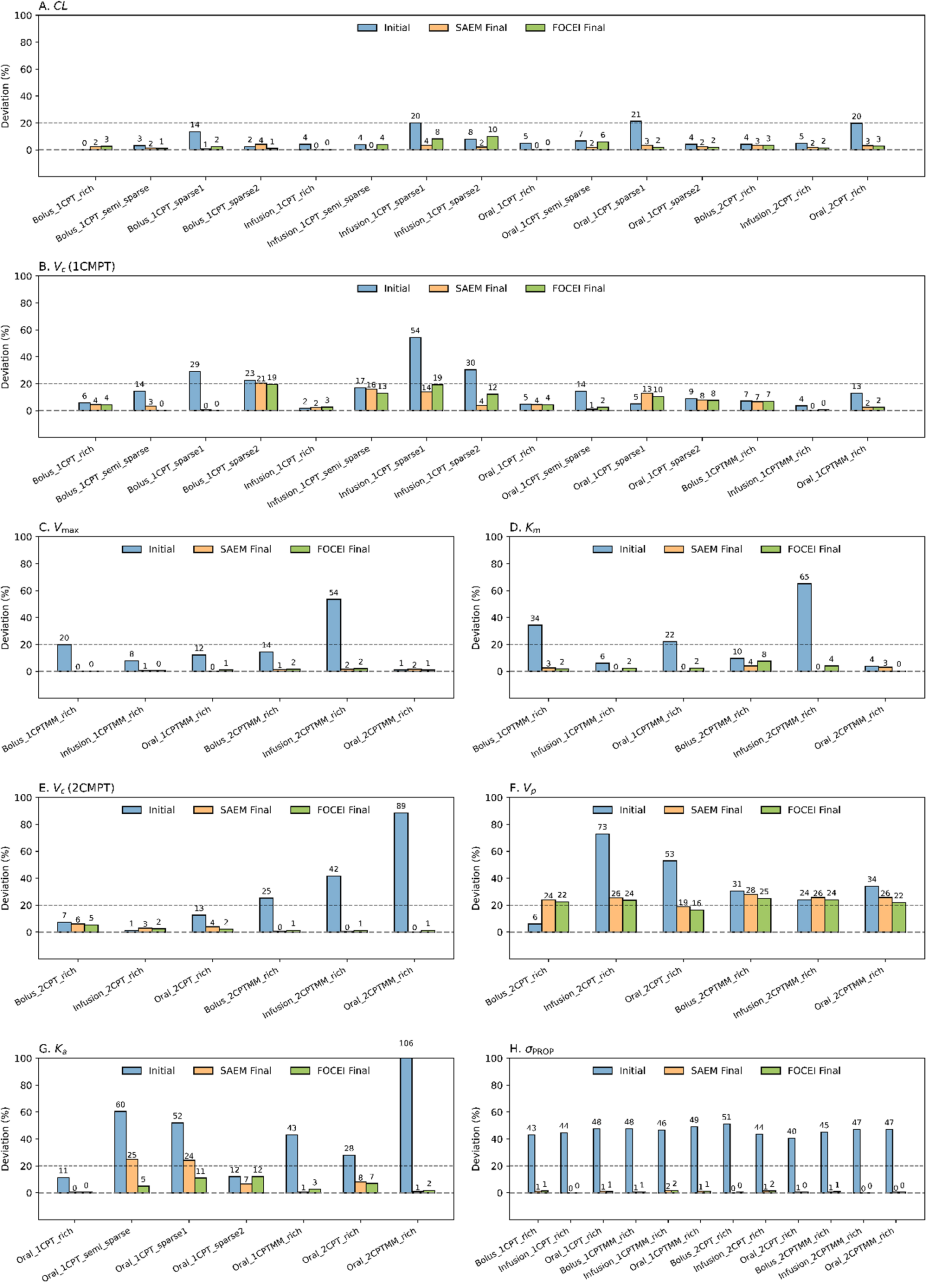
Initial and final estimate deviations [%] from true values used in the simulation across simulated datasets. Each subplot corresponds to one PK parameter: (**A**) CL, clearance, (**B**) V_c_ (1CMPT), the central volume of distribution in a one-compartment model, (**C**) V_max_, the maximum metabolic rate, (**D**) K_m_, the Michaelis-Menten constant, (**E**) V_c_ (2CMPT), the central volume in a two-compartment model, and (**F**) V_p_, the peripheral volume of distribution. (**G**) K_a_ absorption rate constant, (**H**) standard deviations of proportional residual error model. Bars represent the percentage deviations of parameter estimates from their true values, with blue bars indicating the initial estimate deviations (before model fitting), orange bars showing the final deviations after fitting with the SAEM algorithm, and green bars showing the final deviations after fitting with the FOCEI algorithm. A dashed black horizontal Line at 20% denotes a reference threshold

For CL (Fig. 3A), all final estimates obtained via model re-estimation using SAEM and FOCEI with pipeline-recommended initial values were within ± 20% of the true values across all 15 datasets, with a maximum deviation of 10%. Final estimates of V_d_ in the one-compartment model (referred to as V_c_ (1CMPT) in Fig. 3B) using FOCEI with pipeline-recommended initial values were within ± 20% of the true values in all datasets. For SAEM, all but one dataset met this criterion, with *Bolus_1CPT_sparse2* deviating by approximately 21%. Final estimates of K_a_ using SAEM and FOCEI were within ± 30% of the true values, with all FOCEI estimates within ± 20% and two SAEM-based estimates in non-rich datasets deviating by 24% and 25% (Fig. 3G). Initial estimates for CL deviated from the true values by 0–21%. For V_c_, deviations ranged from 0 to 54%, with values exceeding 20% observed primarily in sparse datasets. For K_a_, deviations ranged from 0 to 106%, with the largest deviation observed in a two-compartment nonlinear case.

In addition to summarizing the final output values of CL, V_d_, and K_a_, the results from each candidate pipeline method (adaptive single-point method, naïve pooled NCA, and graphical methods) were compared across rich, semi-sparse, and sparse datasets as presented in Supplementary Table 2 (Material 2). Across the three rich datasets, the pipeline consistently selected the initial estimates from naïve pooled NCA as the recommended output, though all three candidate methods yielded identical values for their final estimates. Similarly, in two intravenous semi-sparse datasets, naïve pooled NCA was selected, with final estimates differing by less than 1% from those of the adaptive single-point method. In both *semi-sparse1* and *sparse1* oral datasets, the adaptive single-point method was the only candidate that produced valid estimates, and selected by the pipeline. In *sparse2* oral dataset, the graphic methods were the only approach that successfully provided the three values that achieved convergence in parameter re-estimation. 

### Pipeline output - extended Pharmacokinetic parameters and residual error

Deviations were evaluated between initial estimates obtained through parameter sweeping and the corresponding final estimates derived using these initial estimates across 12 cases originating from either Michaelis-Menten elimination or a two-compartment model. Values of V_max_ and K_m_ were proposed by the pipeline as shown in Supplementary Tables 3 and 4 (Material 2). From Fig. 3C and Fig. 3D, both V_max_ and K_m_ successfully achieved convergence during re-estimation across six simulated datasets using SAEM or FOCEI methods. The re-estimated V_max_ and K_m_ values were within 2% (984 to 1020 mg/h) and 10% (231 to 258 mg/L) of the true values. The initial estimates of V_max_ and K_m_ selected by the pipeline deviated from the final estimates by less than twofold for all six test datasets.

Pipeline-proposed initial estimates successfully enabled final estimates to converge to true values in the cases of the two-compartment parameters. From Fig. 3E and Fig. 3F, V_c_ successfully achieved convergence during re-estimation across six simulated datasets using SAEM or FOCEI methods, while V_p_ showed partial deviations ranging from 20 to 30%, and all final estimated values between 46.5 and 51.2 L. It remained within a reasonable range compared with the original value of 40 L. The initial estimates of V_c_ and V_p_ selected by the pipeline deviated from the final estimates by less than twofold for all six test datasets.

For residual error model components (Fig. 3H**)**, the final estimates of the residual standard deviation deviated by less than 2% from the true value (0.2) in rich datasets, while the initial estimates deviated by approximately 43–51% across rich datasets. In semi-sparse and sparse datasets, the pipeline applied a fallback value of 0.2 for the residual error parameter; this value was not estimated from the data and did not contribute to the final statistics.

### Pipeline performance- comparison with other strategies in simulated datasets

Parameter re-estimation results across 21 simulated datasets through five initial estimate strategies *(inits = 1*,* nls*,* nlm*,* nlminb*,* pipeline*) were reported in Supplementary Tables 3 and 4 (Material 2). The statistics of final estimates’ deviations from true values for all 21 cases are shown in Supplemental Tables 5 and 6 (Material 2). Overall, for FOCEI, the pipeline achieved final estimates of model structural parameters within a 20% range for 16 (76%) of 21 cases. This percentage increased to 100% when the threshold was expanded to 30%. For SAEM, 13 cases had final estimates within this 20% range. Apart from the pipeline strategy, fewer than half of the cases using other strategies had all final estimates falling within 30% of the true values.

**Fig. 4:**
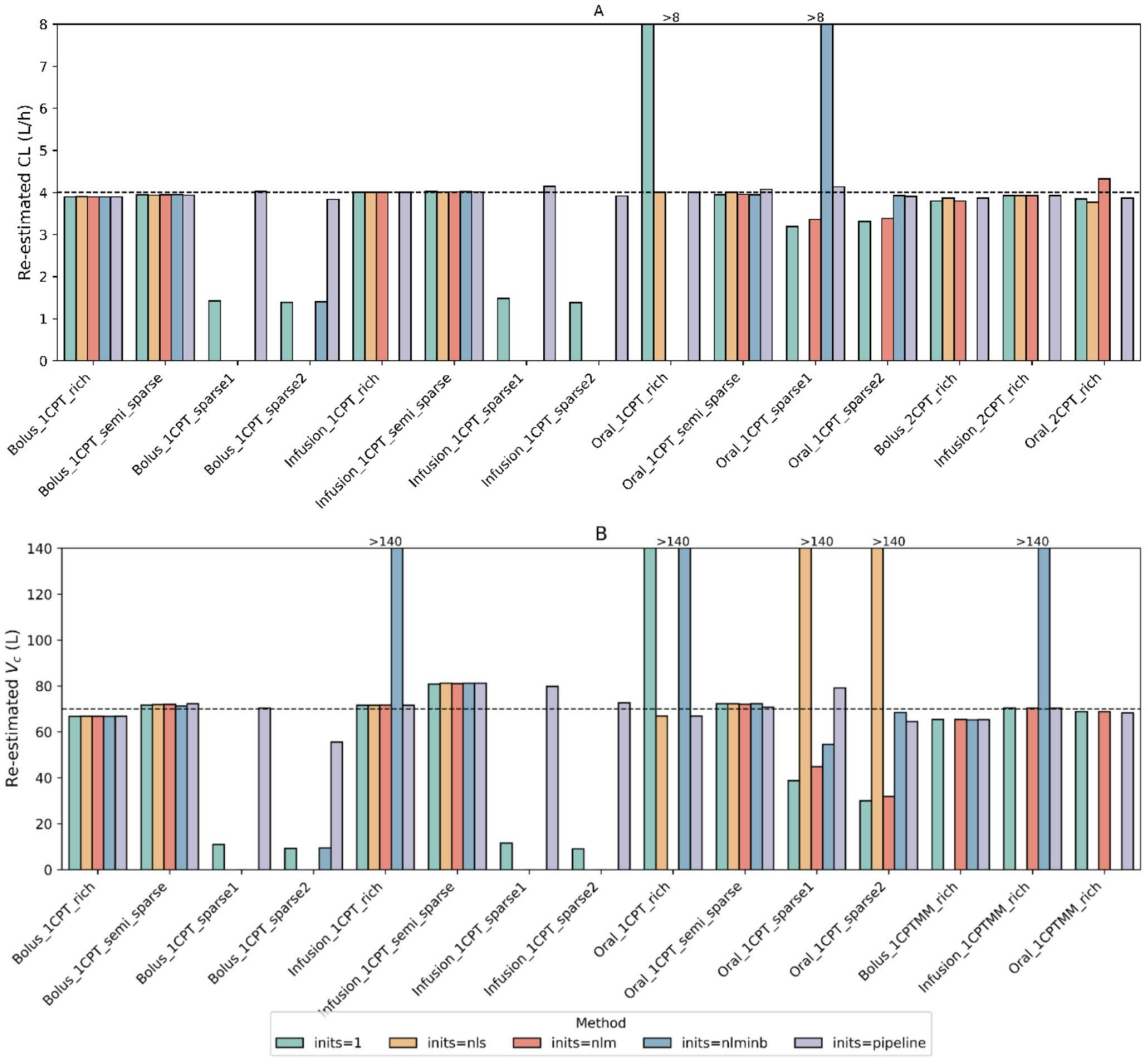
Comparison of re-estimated clearance (top) and volume of distribution (bottom) in simulated datasets across different strategies of setting initial estimates run by SAEM. This figure containes re-estimation of clearance and volume of distribution using five different initial estimate strategies, represented by distinct colors. *inits = 1* sets all initial estimates to 1, while *inits = nls*, *inits = nlm*, and *inits = nlminb* used parameter estimates from respective algorithms as initial values. *inits = pipeline* referred to pipeline-specific recommendations. To address excessively large initial estimates, the y-axis was capped at 2-fold of the true values. Bars exceeding this limit were truncated at the 2-fold value and annotated with “>2-fold” to indicate their magnitude

Comparative final estimate results for CL and V_c_ using different strategies in tests of 15 linear elimination cases and 15 one-compartment cases were highlighted in Fig. 4. Only the pipeline consistently achieved final estimates of CL and V_c_ close to the true values in all cases. In contrast, other strategies had instances of either failing to produce estimates or resulting in overestimations. For CL, while the pipeline achieved 100% success rate, strategies using *inits = 1*, *inits = nls*, and *inits = nlm* as initial estimates achieved only 9 to 10 (60–67%) successful cases under SAEM approach. For V_c_ in the one-compartment model, the pipeline succeeded in 14 of 15 cases, while the figures ranged from 6 to 8 cases for other strategies, approximately half as many as the pipeline. Under the FOCEI approach, the pipeline achieved 100% success in producing estimates within 20% deviation across all key parameters. In contrast, other initialization strategies showed much lower success rates, ranging from 0 to 60% for CL and 5%−38% for V_c_.

For model-specific parameters in more complex models, the pipeline was the only method that achieved final parameter estimates of both V_max_ and K_m_ within 20% of the true values regardless of whether SAEM or FOCEI was used in six test cases. For the remaining strategies, *inits = 1* and *inits = nlm* worked in three one-compartment cases run by the SAEM. Using *inits = nls* and *inits = nlminb* succeeded in only 0–1 cases, and all of them showed similarly limited performance, with no more than 0–1 successful cases under FOCEI. For V_c_ and V_p_ parameters, the pipeline method remained the best approach with all estimated V_c_ within a 20% range, although five cases of V_p_ deviated by 22–28% from the true values. Under the 20% criterion, none of the remaining strategies successfully estimated both V_c_ and V_p_. However, under the 30% criterion, three linear cases using *inits = 1* managed to simultaneously converge both V_c_ and V_p_ to within 30% of the true values in SAEM runs. This was followed by two cases using *inits = nls* and *inits = nlm*, while *inits = nlminb* had no successful cases.

### Pipeline performance- comparison with other strategies in clinical trial datasets

The results of testing the pipeline and the *inits = 1* strategy in both one- and two-compartment models using FOCEI are presented in Table 2. In general, the pipeline outperformed the strategy of *inits = 1*. When parameter estimation was performed using the one-compartment model, both methods produced identical or highly similar parameter estimates in 12 out of 13 cases, with differences within the 20% range. The only exception was the aprindine dataset, where the strategy of *inits = 1* resulted in a final estimate of V_c_ of 4.91 L, whereas the pipeline strategy yielded a final estimate of 271 L. However, the former’s relative standard error (RSE) was much higher than the latter’s (35.5% vs. 1.04%).

**Table 2:** Comparison of parameter estimation results using initial values set to 1 vs. pipeline recommendations for one- and two-compartment models (FOCEI)

Dataset	inits = 1 (1cmpt_fo)	inits = 1 (2cmpt_fo)	inits = pipeline (1cmpt_fo)	inits = pipeline (2cmpt_fo)	PK reference value from source
pheno_sd	CL = 0.00587 [1.54] ^a^ V_c_ = 1.44 [15.7] add = 2.61 prop = 0.0417 AIC = 1022 Run time = 0.0733 min	CL = 2.72 [8.1e + 04] V_c_ = 0.509 [295] V_p_ = 2.72 [8.1e + 04] Q = 2.72 [8.1e + 04] add = 26.4 prop = 7.07e-07 AIC = 1652 Run time = 0.17 min	CL = 0.00589 [1.55] V_c_ = 1.45 [15.6] add = 2.64 prop = 0.0379 AIC = 1022 Run time = 0.0747 min	CL = 0.0057 [1.62] V_c_ = 0.816 [70.6] V_p_ = 0.587 [25.6] Q = 0.515 [20.3] add = 2.84 prop = 0.0072 AIC = 1020 Run time = 2.27 min	CL = **0.0047** L/h/kg V_d_ = **0.96** L/kg [34]
theo_sd	K_a_ = 1.48 [57.7] CL/F = 2.79 [7.69] V_c_/F = 32 [1.52] add = 0.276 prop = 0.134 AIC = 363 Run time = 0.0911 min	K_a_ = 1.31 [72.6] CL/F = 2.75 [7.29] V_c_/F = 28.9 [1.85] V_p_/F = 3.29 [36] Q/F = 1.68 [107] add = 0.281 prop = 0.13 AIC = 369 Run time = 0.261 min	K_a_ = 1.47 [50.2] CL/F = 2.79 [6.14] V_c_/F = 32 [1.3] add = 0.247 prop = 0.137 AIC = 363 Run time = 0.101 min	K_a_ = 1.3 [93.2] CL/F = 2.75 [7.51] V_c_/F = 28.7 [4.16] V_p_/F = 3.52 [53.9] Q/F = 1.8 [141] add = 0.278 prop = 0.131 AIC = 369 Run time = 0.247 min	
theo_md	K_a_ = 1.38 [49.1] CL/F = 2.85 [6.51] V_c_/F = 31.5 [1.17] add = 0.663 prop = 0.138 AIC = 848 Run time = 0.22 min	K_a_ = 1.26 [67.2] CL/F = 2.83 [5.97] V_c_/F = 29.4 [1.04] V_p_/F = 2.24 [44.3] Q/F = 1.35 [160] add = 0.657 prop = 0.139 AIC = 855 Run time = 1.2 min	K_a_ = 1.37 [49] CL/F = 2.84 [6.07] V_c_/F = 31.4 [1.21] add = 0.667 prop = 0.138 AIC = 848 Run time = 0.229 min	K_a_ = 1.27 [128] CL/F = 2.5 [132] V_c_/F = 30.4 [7.98] V_p_/F = 114 [211] Q/F = 0.585 [643] add = 0.714 prop = 0.124 AIC = 850 Run time = 0.793 min	
aprindine	K_a_ = 0.00734 [7.85] CL/F = 1.82 [51.2] V_c_/F = 4.91 [35.5] add = 0.153 prop = 0.283 AIC = 288 Run time = 0.607 min	K_a_ = 1.73e + 03 [3.12] CL/F = 0.00806 [15] V_c_/F = 0.0399 [11.2] V_p_/F = 380 [1.17] Q/F = 1.95 [77.9] add = 0.308 prop = 0.268 AIC = 392 Run time = 7.28 min	K_a_ = 0.431 [24.1] CL/F = 1.35 [74] V_c_/F = 271 [1.04] add = 0.145 prop = 0.282 AIC = 288 Run time = 0.12 min	K_a_ = 0.42 [37.9] CL/F = 1.28 [338] V_c_/F = 269 [2.51] V_p_/F = 51.6 [618] Q/F = 0.109 [582] add = 0.153 prop = 0.283 AIC = 296 Run time = 0.483 min	
cefaclor	K_a_ = 1.5 [25.3] CL/F = 32.4 [1.65] V_c_/F = 23.4 [1.43] add = 0.001 prop = 0.454 AIC = 696 Run time = 0.457 min	K_a_ = 1.49 [21.9] CL/F = 32.4 [1.84] V_c_/F = 22.8 [2.75] V_p_/F = 0.389 [102] Q/F = 23.3 [19.1] add = 0.001 prop = 0.452 AIC = 704 Run time = 6.46 min	Ka = 1.53 [24.6] CL/F = 32.4 [1.71] V_c_/F = 23.8 [1.57] add = 0.001 prop = 0.452 AIC = 696 Run time = 0.364 min	Ka = 1.5 [21.2] CL/F = 32.3 [1.74] V_c_/F = 23.3 [1.5] V_p_/F = 12.4 [27.2] Q/F = 0.166 [15.1] add = 0.001 prop = 0.452 AIC = 705 Run time = 1.65 min	
ceftriaxone	CL/F = 0.159 [12.4] V_c_/F = 1.27 [75.9] add = 6.93 prop = 0.199 AIC = 696 Run time = 0.0173 min	CL/F = 0.127 [20.6] V_c_/F = 0.463 [53.2] V_p_/F = 0.881 [360] Q/F = 0.661 [90.9] add = 1.13 prop = 0.128 AIC = 709 Run time = 0.158 min	CL = 0.159 [12.4] V_c_ = 1.28 [73.7] add = 6.95 prop = 0.197 AIC = 696 Run time = 0.0157 min	CL = 0.2 [39.7] V_c_ = 1.09 [1.92e + 03] V_p_ = 1.42 [315] Q = 0.35 [370] add = 30.2 prop = 0.211 AIC = 717 Run time = 0.0669 min	CL/F = **0.08** L/h at 3.8 kg V_d_/F = **1.71** L at 3.8 kg [35]
cephalexin	K_a_ = 0.874 [78.8] CL/F = 16.7 [1.2] V_c_/F = 9.19 [9.77] add = 0.001 prop = 0.431 AIC = 1017 Run time = 0.511 min	K_a_ = 1.42 [55.8] CL/F = 15.7 [1.49] V_c_/F = 14.5 [5.93] V_p_/F = 24.5 [16.3] Q/F = 2.42 [32.5] add = 0.001 prop = 0.425 AIC = 1008 Run time = 2.04 min	K_a_ = 1.79 [23.5] CL/F = 16.7 [1.17] V_c_/F = 18.4 [2.19] add = 0.001 prop = 0.436 AIC = 1011 Run time = 0.42 min	K_a_ = 1.45 [47.6] CL/F = 15.7 [1.65] V_c_/F = 14.9 [5.41] V_p_/F = 24.7 [17.5] Q/F = 2.35 [32.9] add = 0.001 prop = 0.425 AIC = 1008 Run time = 2.31 min	
diazepam	CL = 5.09 [10.4] V_c_ = 23.6 [3.11] add = 0.0517 prop = 0.21 AIC = −272 Run time = 0.0159 min	CL = 2.74 [15.2] V_c_ = 15.4 [5.42] V_p_ = 24.9 [6.88] Q = 14.4 [8.86] add = 0.00589 prop = 0.185 AIC = −426 Run time = 0.195 min	CL = 4.24 [13.4] V_c_ = 26.6 [2.86] add = 0.0292 prop = 0.297 AIC = −281 Run time = 0.0105 min	CL = 2.59 [10.8] V_c_ = 16.3 [4.69] V_p_ = 26.5 [6.13] Q = 10.4 [6.17] add = 0.00561 prop = 0.179 AIC = −429 Run time = 0.0683 min	CL (derived) ^b^ = **3.09** L/h V_c_ (derived) = **24.11** L V_p_ (derived) = **18.71** L V_p2_ (derived) = **73.83** L Q (derived) = **56.23** L/h Q_2_ (derived) = **8.84** L/h [36]
fluorouracil	CL = 66.5 [3.51] V_c_ = 12.7 [7.25] add = 0.101 prop = 0.318 AIC = 349 Run time = 0.0117 min	CL = 61.8 [3.62] V_c_ = 8.97 [12.5] V_p_ = 2.08 [42.4] Q = 13.9 [14.5] add = 0.0903 prop = 0.278 AIC = 349 Run time = 0.071 min	CL = 67.1 [3.51] V_c_ = 12.6 [7.29] add = 0.0947 prop = 0.321 AIC = 349 Run time = 0.0112 min	CL = 53.4 [19.4] V_c_ = 11.1 [16.1] V_p_ = 92.5 [124] Q = 12.5 [102] add = 0.0985 prop = 0.309 AIC = 353 Run time = 0.0481 min	CL (derived) = **86.5 L/h** V_c_ = **13.1** L [37] CL = **75.9** L/h V_c_ = **20.3** L [38]
oxprenolol (iv)	CL = 24 [1.68] V_c_ = 43.4 [0.757] add = 7.07 prop = 0.164 AIC = 948 Run time = 0.0134 min	CL = 24.1 [1.63] V_c_ = 3.17 [54.1] V_p_ = 42.8 [1.39] Q = 813 [5.71] add = 5.26 prop = 0.128 AIC = 924 Run time = 0.438 min	CL = 24.1 [1.68] V_c_ = 43.4 [0.756] add = 7.3 prop = 0.162 AIC = 948 Run time = 0.0168 min	CL = 23 [1.49] V_c_ = 33.7 [0.866] V_p_ = 18.6 [5.77] Q = 20.2 [5.73] add = 2.58 prop = 0.0547 AIC = 819 Run time = 0.113 min	CL (derived) = **23.56** L/h V_c_ = **34.4** L V_p_ (derived) = **16.39** L Q (derived) = **23.6** L/h [39]
oxprenolol (oral)	K_a_ = 2.23 [16.5] CL/F = 60.3 [1.53] V_c_/F = 139 [1.26] add = 1.03 prop = 0.342 AIC = 2243 Run time = 0.0721 min	K_a_ = 62.9 [1.37] CL/F = 14.5 [2.11] V_c_/F = 0.257 [9.27] V_p_/F = 9.36 [4.65] Q/F = 4.88 [7.34] add = 0.836 prop = 0.392 AIC = 2294 Run time = 0.671 min	K_a_ = 2.35 [15.7] CL/F = 60.6 [1.54] V_c_/F = 142 [1.26] add = 0.001 prop = 0.346 AIC = 2242 Run time = 0.0868 min	K_a_ = 0.918 [79.1] CL/F = 58.9 [1.47] V_c_/F = 61.8 [2.34] V_p_/F = 56.7 [5.12] Q/F = 20.9 [4.2] add = 0.001 prop = 0.322 AIC = 2217 Run time = 0.44 min	F_bio_ (mean) = 0.43 CL (derived) = **54.8** L/h V_c_ (derived) = **80** L V_p_ (derived) = **38.1** L Q (derived) = **54.9** L/h [39]
pindolol	K_a_ = 1.28 [97.5] CL/F = 24.8 [4.44] V_c_/F = 110 [2.2] add = 1.33 prop = 0.187 AIC = 649 Run time = 0.0396 min	K_a_ = 22.7 [4.46] CL/F = 7.32 [4.04] V_c_/F = 0.127 [76.9] V_p_/F = 13.8 [1.76] Q/F = 3.36 [2.32] add = 0.779 prop = 0.322 AIC = 683 Run time = 0.639 min	K_a_ = 1.31 [98.1] CL/F = 24.8 [3.85] V_c_/F = 111 [2.21] add = 1.32 prop = 0.187 AIC = 649 Run time = 0.0337 min	K_a_ = 0.988 [1.99e + 03] CL/F = 23.8 [3.9] V_c_/F = 87.5 [2.47] V_p_/F = 29.5 [14.1] Q/F = 10.7 [11.7] add = 1.05 prop = 0.192 AIC = 652 Run time = 0.188 min	CL = **25.5** L/h V_c_ =**142** L [40]
tobramycin	CL = 4.03 [4.33] V_c_ = 24.8 [1.51] add = 0.001 prop = 0.261 AIC = 788 Run time = 0.786 min	CL = 3.57 [4.41] V_c_ = 12.4 [10.7] V_p_ = 8.06 [12.9] Q = 3.37 [31.7] add = 0.001 prop = 0.255 AIC = 848 Run time = 6.5 min	CL = 4.44 [4.18] V_c_ = 25.4 [1.69] add = 0.001 prop = 0.274 AIC = 798 Run time = 0.407 min	CL = 3.83 [31] V_c_ = 21.5 [0.809] V_p_ = 7 [9.84] Q = 0.365 [64] add = 0.001 prop = 0.256 AIC = 756 Run time = 3.05 min	CL (derived) = **3.8** L/h V_c_ (derived) = **21.8** L Q (derived) = **0.26** L/h V_p_ (derived) = **9.6** L [41]

Abbreviations: 1cmpt_fo, a one-compartment model with first-order elimination (or first-order absorption and elimination in oral cases); 2cmpt_fo, a two-compartment model with first-order elimination (or first-order absorption and elimination in oral cases); Run time: computational running time; add, additive residual error; prop, proportional residual error.

^a^ Parameter estimates are presented as typical population estimates with their corresponding relative standard errors (RSE%) indicated in brackets. Except for the pheno_sd case, where the unit of CL is L/h/kg and the unit of V is L/kg, the units of CL and V in all other cases are L/h and L, respectively.

^b^ Parameter (derived) refers to the value that was not explicitly reported in the original reference but was calculated based on other reported parameters. The following formulas were applied when calculating parameters: k_12_ = Q/V_c_, k_21_ = Q/V_p_, and k_el_ = CL/V_c_, k_13_ = Q_2_/V_c_, k_31_ = Q_2_/Vp_2_, Here, k_12_ and k_13_ represent the rate constant describing the transfer of the drug from the central compartment to the peripheral compartment and second peripheral compartment, k_21_ and k_31_ is the rate constant for the transfer of the drug from the peripheral compartment and second peripheral compartment to the central compartment, and k_el_ is the elimination rate constant. For parameters with covariate models, calculation was based on median covariate values. In the case of diazepam which reported parameters of four individuals, parameter values were summarized using the geometric mean.

*Inits = 1* performed worse than the pipeline when running a one-compartment model by SAEM (Supplementary Table 7, Material 2). Three cases, aprindine, cefaclor, and ceftriaxone, showed substantially different parameter estimation results. *Inits = 1* and pipeline resulted in CL values of 102 vs. 1.52, 3350 vs. 30.9, and 225 vs. 0.329 L/h, respectively. Similarly, for V_c_, the estimates were 0.0149 vs. 263, 508 vs. 22.8, and 252 vs. 1.54 L. Notably, the three AIC values resulting from the *inits = 1* strategy were found to be much larger than those from the pipeline, with differences ranging from 2- to 3-fold. And in these three cases, RSE% values exceeded 1000% for CL and V_c_ estimates using *inits = 1*, except for cefaclor CL (142%). In contrast, the RSE values based on the pipeline strategy ranged from 1.38 to 129%. Regarding computational time run by SAEM and FOCEI, both strategies were completed within one minute, as shown in Supplementary Figs. 4 and 5 (Material 2).

Despite the fact that some real-life data might not originate from a two-compartment model, which could increase the inaccuracies in parameter estimates due to the nature of the data. However, two strategies still differed in both goodness-of-fit and computational time. For model fitting performance, there were 8 of 13 cases where the pipeline method’s AIC was lower than that using *inits = 1* run by FOCEI. The remaining five cases showed either identical or similar AIC performance between the two strategies. In 10 of 13 cases, AIC values for the pipeline were lower than *inits = 1* when running by SAEM. In the remaining three cases (fluorouracil, pindolol, and diazepam), RSE% values of CL and V_c_ using the pipeline were all less than 20%. However, when using the *inits = 1* strategy, two cases exhibited substantially higher RSE% for CL or V_c_, ranging from 32.8 to 853%, with the only exception being fluorouracil CL, which had a value of 2.59%. For the last case, diazepam, the parameter estimates between the two methods differed by less than 2%.

Regarding time spent running with two-compartment models, the pipeline took 3–40 s, while it was 21–105 s for *inits = 1* strategy. FOCEI followed this trend, with all cases using the pipeline taking less than 4 min. In contrast, the longest times among the three occurred with the *inits = 1* method, ranging from 6.5 to 7.3 min.

## Discussion

In this study, an automated pipeline was developed to generate initial estimates for structural and statistical parameters in PopPK modeling, applicable to both rich and sparse data as well as for intravenous and extravascular route of administration. This pipeline is particularly useful in the absence of *a priori* information or when no iterative optimization has yet been performed, providing a reasonable starting point for the first round of modeling. Assessment results showed that the pipeline performed well both based on simulated and real-life clinical datasets, and that the initial estimates it generates enable reliable and accurate fitting in subsequent re-estimation or model development.

In the pipeline design, data are pooled for NCA and graphic analysis. Compared to the two-stage approach, which first calculates or estimates PK parameters for each individual and then summarizes them at the population level [42], the naïve pooled method is more appropriate for sparse designs with staggered sampling schedules, as it does not rely on complete individual profiles. All three approaches demonstrated their respective strengths during the evaluation. Results from Supplementary Table 2 (Material 2) indicate that the designed adaptive single-point method works in most cases, NCA handles rich data effectively, and graphic methods can address sparse oral data, fulfilling the design goal of flexible adaptation across PK scenarios. In the designed pipeline, the rRMSE, scaled by the pointwise average of predicted and observed values to scale the error, was used as the primary metric. Alternative predictive metrics such as mean absolute percentage error and rRMSE (i.e., using the mean of observed values as denominator) are also supported by the pipeline. However, exploratory analysis (Supplementary Fig. 6, Material 2) showed these metrics tended to overestimate nonlinear parameters like V_max_ and K_m_, possibly due to stronger penalization of prediction bias.

For pipeline performance in the simulated datasets, some non-clearance parameters showed 20–30% deviation in final estimates, likely due to data sparsity or suboptimal design causing model unidentifiability, though a lenient 30% threshold still yielded 100% overall success. Another exploratory analysis in this study (Supplementary Table 8, Material 3), which re-estimated the models using the true values as initial estimates, did not reduce the objective function values, suggesting that the pipeline had already converged to optimal or near-optimal solutions. Two types of initialization strategies were compared with the pipeline. The *inits = 1* strategy mimicked model fitting without *a priori* knowledge or user intervention. The other, algorithm-optimized strategy has been used in practice, for example, in Monolix [43], where parameters are initially set to 1 and subsequently optimized internally. Results illustrated that the pipeline outperformed other initialization strategies implemented in this study. *Inits = 1* led to successful parameter convergence in 47.6% of cases, and this number dropped to 0 when using FOCEI, with most estimates failing to move from the initial setting during estimation. This finding aligns with previous literature suggesting that SAEM generally provides better estimates than FOCEI [44, 45], and it could be particularly true when initial estimates are extremely poor. Another observation from this study was that using methods (nls, nlm, and nlminb) to optimize initial estimates was far less effective than directly following pipeline recommendations. One possible reason is nonlinear parameter estimation is highly sensitive to initial estimates [33]. Therefore, it may be necessary to provide initial guesses for the estimates or introduce boundary constraints.

For real-life data, the pipeline consistently demonstrated superior performance compared to the *inits = 1* strategy, which showed the potential to cause severe parameter estimation bias. In the aprindine case, the *inits = 1* strategy led to a final FOCEI V_c_ estimate of 4.95 L (Table 2), which deviated significantly from a previously reported range of 164–351 L [46]. In contrast, the pipeline had a more accurate final value of 271 L. For CL, the *inits = 1* strategy and the pipeline yielded estimates of 0.00806 and 1.35 L/h at multiple doses (200 and 100 mg). Reported values were 50.6 and 13.4 L/h at doses of 50 and 100 mg [46]. Value from the pipeline was more acceptable given the nonlinear kinetics and CL decrease ratio. Furthermore, the pipeline may play a more effective role in facilitating appropriate model selection. For tobramycin, the AIC generated by the pipeline for the two-compartment model (756) was lower than the AIC for the one-compartment model (798), prompting selection of the better-performing two-compartment model, as also suggested in the source reference [41]. In contrast, using the *inits = 1* strategy resulted in an AIC of 848 for the two-compartment model and 788 for the one-compartment model, leading to the opposite selection.

Regarding time efficiency, using pipeline-recommended initial estimates significantly reduced computation time with SAEM or FOCEI compared to *inits = 1*. The time savings can reach up to five-fold applying SAEM or FOCEI when using a two-compartment model. For the time spent by the pipeline itself, the average runtime was approximately 29 s per dataset (range: 16–62 s), depending on model complexity and sampling density (Supplementary Fig. 7, Material 2). In the practice of automated modeling, where thousands of models may be tested [47], excessive runtime on poorly fitted or incorrect models can lead to a substantial waste of both resources and time. Therefore, utilizing the pipeline’s adaptive approach to provide more accurate initial estimates enhances model accuracy and optimizes computational efficiency, making it a valuable tool for large-scale pharmacokinetic modeling.

There are several implicit limitations in this study. The adaptive-single-point method and the graphic methods rely on the assumption of one-compartment with linear kinetics, and their outputs were also tested within the same model. Parameter sweeping for V_max_ and K_m_ was based on the one-compartment model, while multi-compartment parameter sweeping was carried out under the assumption of first-order kinetics. These assumptions may introduce bias when applied to drugs that follow a two-compartment model or exhibit nonlinear absorption or metabolism. Although results from these studies have shown good performance for two-compartment and nonlinear models, further testing is needed to evaluate how these assumptions may impact real-life applications. The pipeline assumes rapid absorption relative to elimination, which may limit its applicability in flip-flop kinetics, such as those seen in extended-release formulations. It may also require further validation for drugs with characteristics, such as very long half-lives (e.g., several months) or very short half-lives (e.g., less than 1 h). Additionally, optimal design can also help assess model identifiability, particularly in settings with sparse or uninformative data, and should be considered in future testing efforts.

The pipeline currently supports the generation of initial estimates for one-compartment model parameters (K_a_, V_d_, and CL), with parameters specific to more complex models, including V_max_, K_m_, V_c_, V_p_, Q, V_p2_, and Q_2_. It also includes strategies for initializing IIV and RUV components. Three-compartment parameters are also available in the pipeline. However, the current study did not include evaluations of three-compartment models, and an assessment of the pipeline’s ability to handle such models is planned as a basis for further applicability extension. The current test datasets are based on the nlmixr2 standard, but the pipeline is expected to support the development of PopPK models in other software programs and to be particularly helpful for beginners who struggle with defining initial estimates. Built as an open-source tool on the nlmixr2 framework, the pipeline generates initial parameter estimates, which can be accessed and exported independently for use in other modeling environments without proprietary software. In addition, the pipeline is highly modular and flexible. For instance, custom values (e.g., final estimates from a one-compartment model) can be specified for the parameter sweeping procedure. While the pipeline does not yet directly link to PopPK software such as NONMEM or Monolix, it has the potential to build an initial estimates bridge in the future through R packages, such as babelmixr2, which can run NONMEM and Monolix within R environment.

In conclusion, the automated pipeline developed in this study is able to not only provide reliable initial estimates for population pharmacokinetic modeling but also proves particularly suitable for scenarios with sparse sampling or lack of *a priori* information. By integrating multiple computational methods, it is a great and promising tool for the provision of initial estimates for both manual and automated modeling applications.

## Supplementary Information

Below is the link to the electronic supplementary material.

ESM 1 (DOCX 1.16 MB)

ESM 2 (DOCX 7.29 MB)

ESM 3 (CSV 3.99 MB)

## Data Availability

Simulated datasets are provided within the supplementary material. Real-life datasets are obtained from published papers.
