# Correction to: An automated pipeline to generate initial estimates for population Pharmacokinetic base models

**DOI:** 10.1007/s10928-025-10017-4

**Published:** 2026-01-04

**Authors:** Zhonghui Huang, Matthew Fidler, Minshi Lan, Iek Leng Cheng, Frank Kloprogge, Joseph F Standing

**Affiliations:** 1 Great Ormond Street Institute of Child Health, University College London, London, UK; 2 Novartis Pharmaceuticals Corporation, Fort Worth, Texas USA; 3 Institute for Global Health, University College London, London, UK; 4 Great Ormond Street Hospital for Children, London, UK

**Correction to: Journal of Pharmacokinetics and Pharmacodynamics (2025) 52:60**

10.1007/s10928-025-10000-z

In this article the author’s name Iek Leng Cheng was incorrectly written as lek Leng Cheng.

The original article has been corrected.

