# Supplementary material for: An automated pipeline to generate initial estimates for population Pharmacokinetic base models
Source: J Pharmacokinet Pharmacodyn. 2025 Nov 6;52(6):60. doi: 10.1007/s10928-025-10000-z (PMC12592298; doi:10.1007/s10928-025-10000-z)
Supplement: Supplementary file 1 — (DOCX 1.16 MB) [file 10928_2025_10000_MOESM1_ESM.docx]

**An automated pipeline to generate initial estimates for population pharmacokinetic base models**

**(Supplemental Material 1)**

Zhonghui Huang^1^, Matthew Fidler^2^, Minshi Lan^1^, lek Leng Cheng^1,4^, Frank Kloprogge^3^, Joseph F Standing^1,4^

^1^Great Ormond Street Institute of Child Health, University College London, London, UK

^2^Novartis Pharmaceuticals Corporation, Fort Worth, Texas, USA

^3^Institute for Global Health, University College London, London, UK

^4^Great Ormond Street Hospital for Children, London, UK

**Corresponding author: Zhonghui Huang**

**Affiliation:** Great Ormond Street Institute of Child Health, University College London, London, UK

**E-mail:** [**zhonghui.huang.20@ucl.ac.uk**](mailto:zhonghui.huang.20@ucl.ac.uk)

**Supplementary Figures**

[**Supplementary Figure 1.** Workflow of data cleaning and pooling in the pipeline 3](#_Toc205284249)

[**Supplementary Figure 2.** Workflow of the adaptive single-point method in the pipeline 4](#_Toc205284250)

[**Supplementary Figure 3.** Workflow of naive pooled NCA and Wanger-Nelson in the pipeline 5](#_Toc205284251)

[**Supplementary Figure 4**. Workflow of graphic methods in the pipeline 6](#_Toc205284252)

[**Supplementary Figure 5.** Workflow of parameter sweeping in the pipeline 7](#_Toc205284253)

[**Supplementary Figure 6.** Workflow of residual error calculation in the pipeline 8](#_Toc205284254)

**Supplementary Tables**

[**Supplementary Table 1.** Summary of thresholds used in the pipeline for steady-state identification 2](#_Toc200340166)

**Supplementary Table 1.** Summary of thresholds used in the pipeline for steady-state identification

| Threshold Label | Threshold Description | Usage Context | Rationale / Assumption | Reference |
| --- | --- | --- | --- | --- |
| Time threshold for approximating steady state | Steady state is considered reached after 5 half-lives | Steady state was approximated when the dosing duration exceeded 5 elimination half-lives | Classic pharmacokinetic principle based on first-order accumulation kinetics: steady state is typically achieved after 5 elimination half-lives, corresponding to >96% of the steady state concentration under first-order kinetics. | [1] |
| Number of Prior Doses for approximate steady state | Five consecutive prior doses used to approximate steady state when the half-life–based estimate exceeds five doses | Used when the number of doses required to reach steady state, based on half-life, exceeds five. | (1) For long half-life drugs, the required number of doses based on half-life may be impractical; (2) Using five prior doses to approximate steady state is a practical and commonly accepted approach and is also consistent with Monolix's default behavior. | [2] |
| Minimum number of documented pre-doses | At least 3 consecutive prior doses are required before the index sample | Used to verify sufficient dosing history | (1) Ensures two full dosing intervals can be evaluated for regularity; (2) As regulatory guidance recommends evaluating ≥3 pre-dose concentrations to confirm steady state, this necessitates that at least three prior doses have been administered at regular intervals. | [3] |
| Dosing interval variability threshold | Dosing intervals must vary by no more than ±25% from the last inter-dose interval before sampling | Used to assess dosing regularity and timing adherence | A ±25% window around the planned dosing interval is widely used in adherence research as the operational definition of “on-time” dosing | [4–6] |
| Dose amount variability threshold | All prior doses must fall within ±25% of the last dose administered before the sample. | Used to evaluate consistency in recent dosing prior to sampling | Assuming linear pharmacokinetics, a ±25% change in dose corresponds approximately to a ±25% change in exposure, aligning with the 80–125% bioequivalence acceptance range. | [3] |

**Supplementary Figure 1.** Workflow of data cleaning and pooling in the pipeline


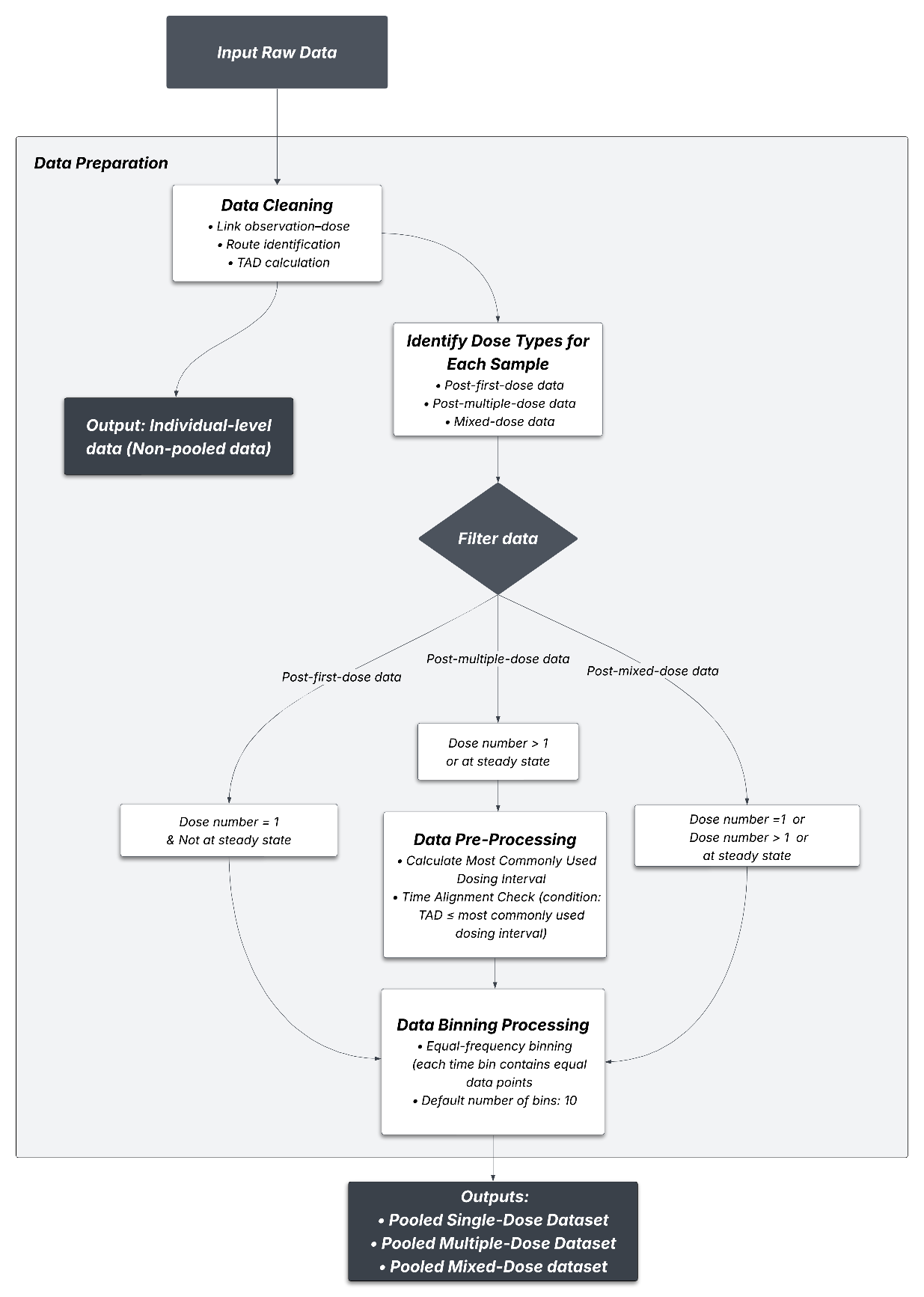


TAD: Time after the last dose

**Supplementary Figure 2.** Workflow of the adaptive single-point method in the pipeline


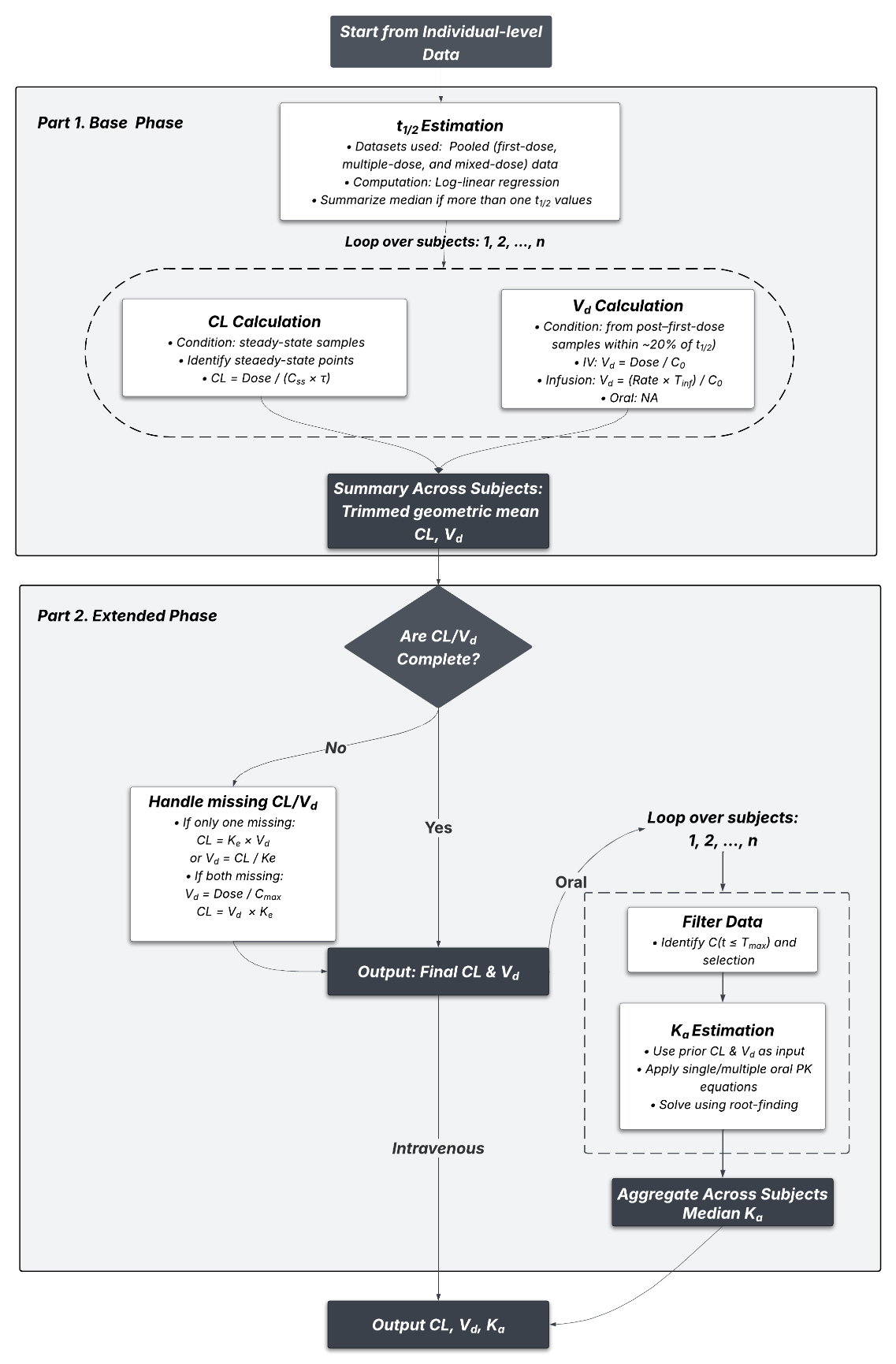


CL: clearance, V_d_: volume of distribution, K_a_: absorption rate constant, t_1/2_: half-life, K_e_: elimination rate, τ: dose interval, T_inf_: time of duration, C_max_: peak time concentration, calculated as mean of each subject peak concentration, C_ss_: concentration at the steady state

**Supplementary Figure 3.** Workflow of naive pooled NCA and Wanger-Nelson in the pipeline


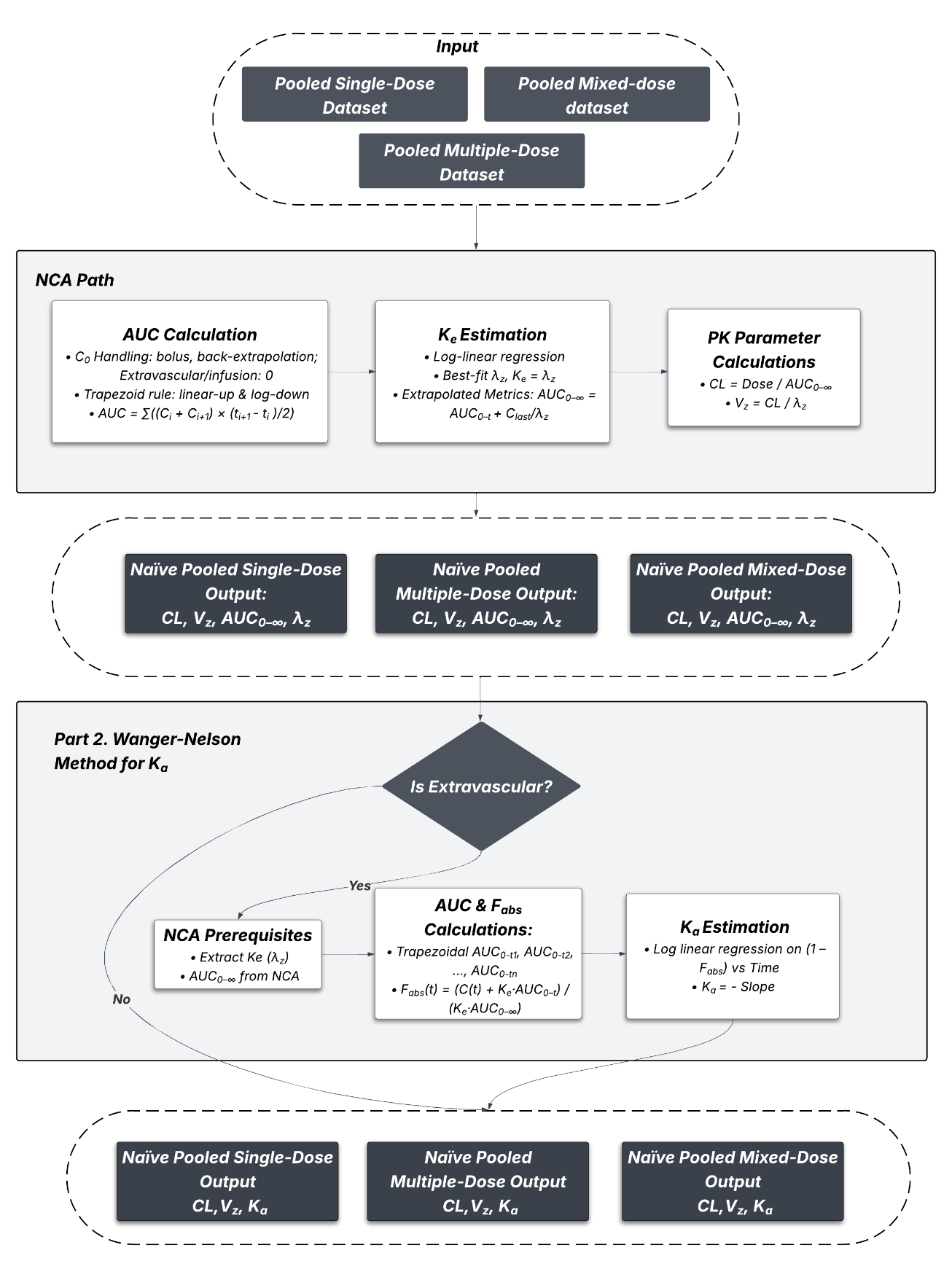


Clast: the last measurable concentration, CL: Clearance, Vz: volume of distribution (based on terminal phase), AUC₀–t: area under the curve from 0 to the t (the last measurable time point), AUC₀–∞: area under the curve extrapolated to infinity, λz: terminal rate constant, Ka: absorption rate constant, Fabs: Fraction absorbed, C0: extrapolated concentration at the time 0, Best-fit λ_z_ : a method to estimate slope of terminal phase. λ_z_ was estimated by iteratively performing log-linear regressions starting from the last three non-zero concentration points and extending backward. The subset with a negative slope and the highest adjusted R-squared was selected as the optimal terminal phase.

**Supplementary Figure 4**. Workflow of graphic methods in the pipeline


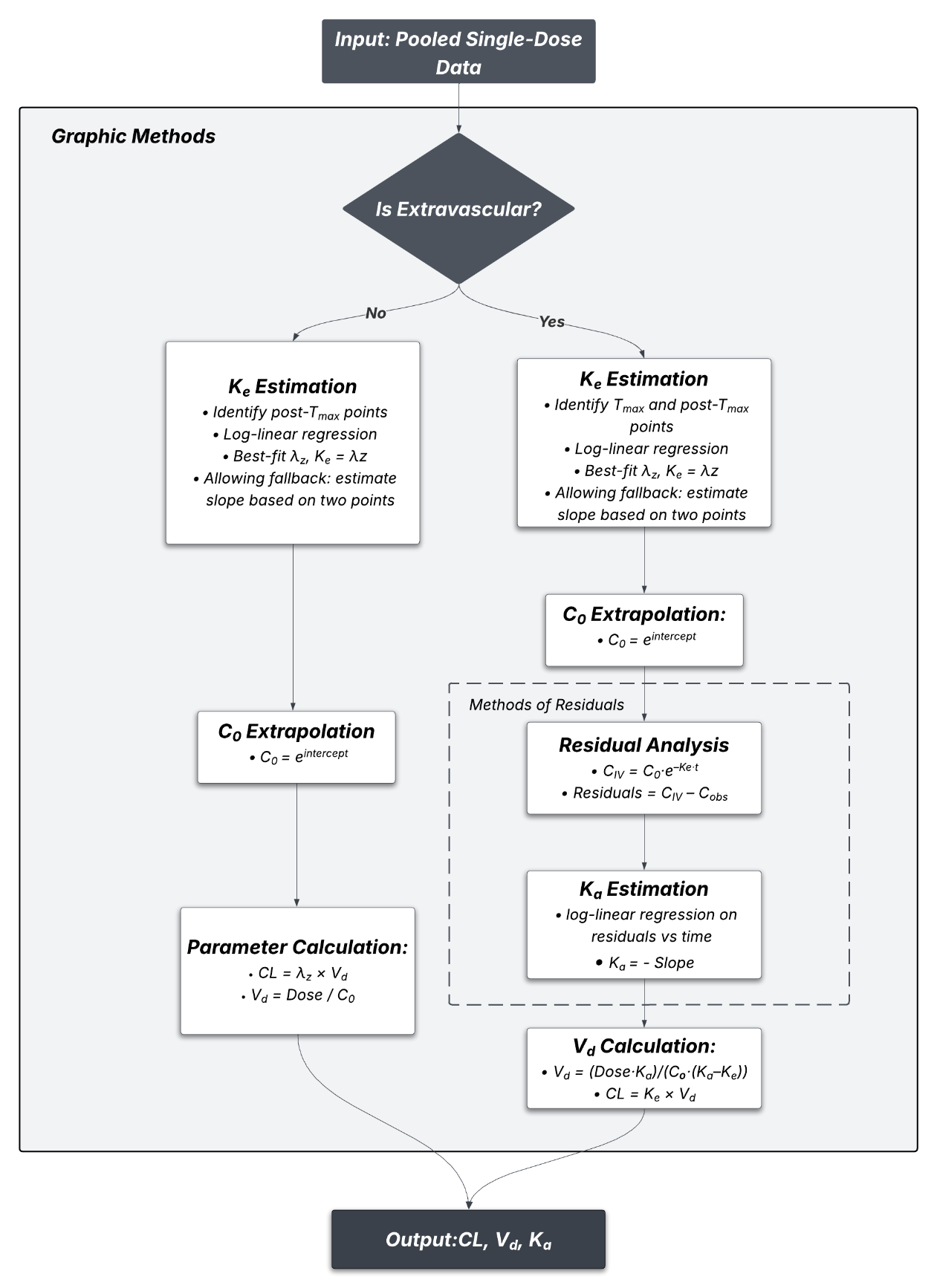


K_e_: elimination rate constant, Ka: absorption rate constant, C_0_: extrapolated concentration at time 0, CL: Clearance, V_d_: volume, λ_z_: Terminal slope, C_IV_: predicted concentration based on intravenous line, C_obs_: observed concentration. Best-fit λz: a method to estimate slope of the terminal phase. λz was estimated by iteratively performing log-linear regressions starting from the last three non-zero concentration points and extending backward. The subset with a negative slope and the highest adjusted R-squared was selected as the optimal terminal phase.

**Supplementary Figure 5.** Workflow of parameter sweeping in the pipeline


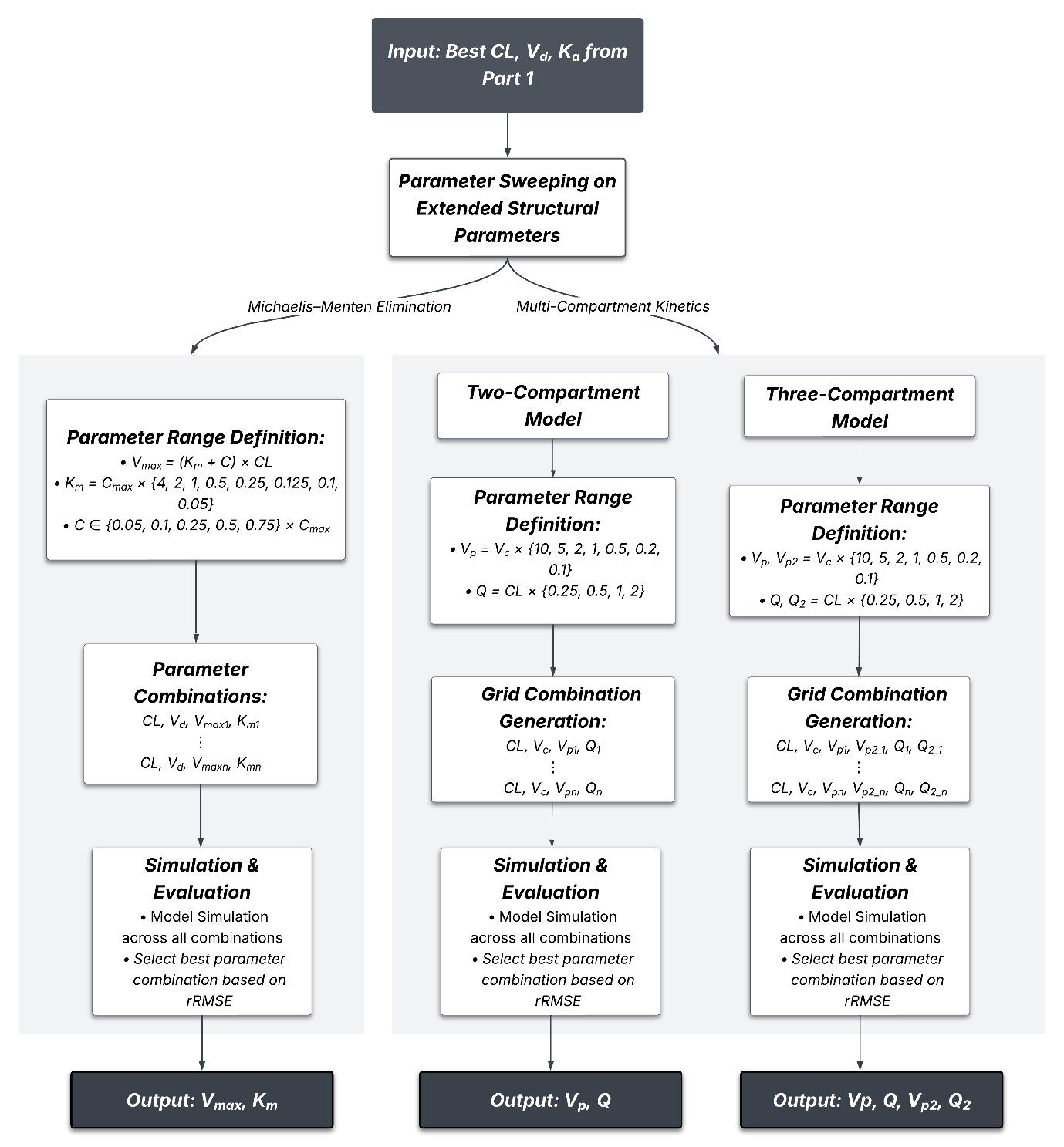


CL: Clearance, V_d_: Volume of distribution, K_a_: Absorption rate constant, V_max_: maximum elimination rate, K_m_: Michaelis constant, C, C_max_: Drug concentration, peak concentration (calculated as mean of each subject peak concentration), V_c_: central volume of distribution, V_p_: volume of distribution of peripheral compartment, Q: inter-compartmental clearance), V_p2_: volume of distribution of the second peripheral compartment), Q_2_: the second inter-compartmental clearance, rRMSE: relative root mean square error

**Supplementary Figure 6.** Workflow of residual error calculation in the pipeline


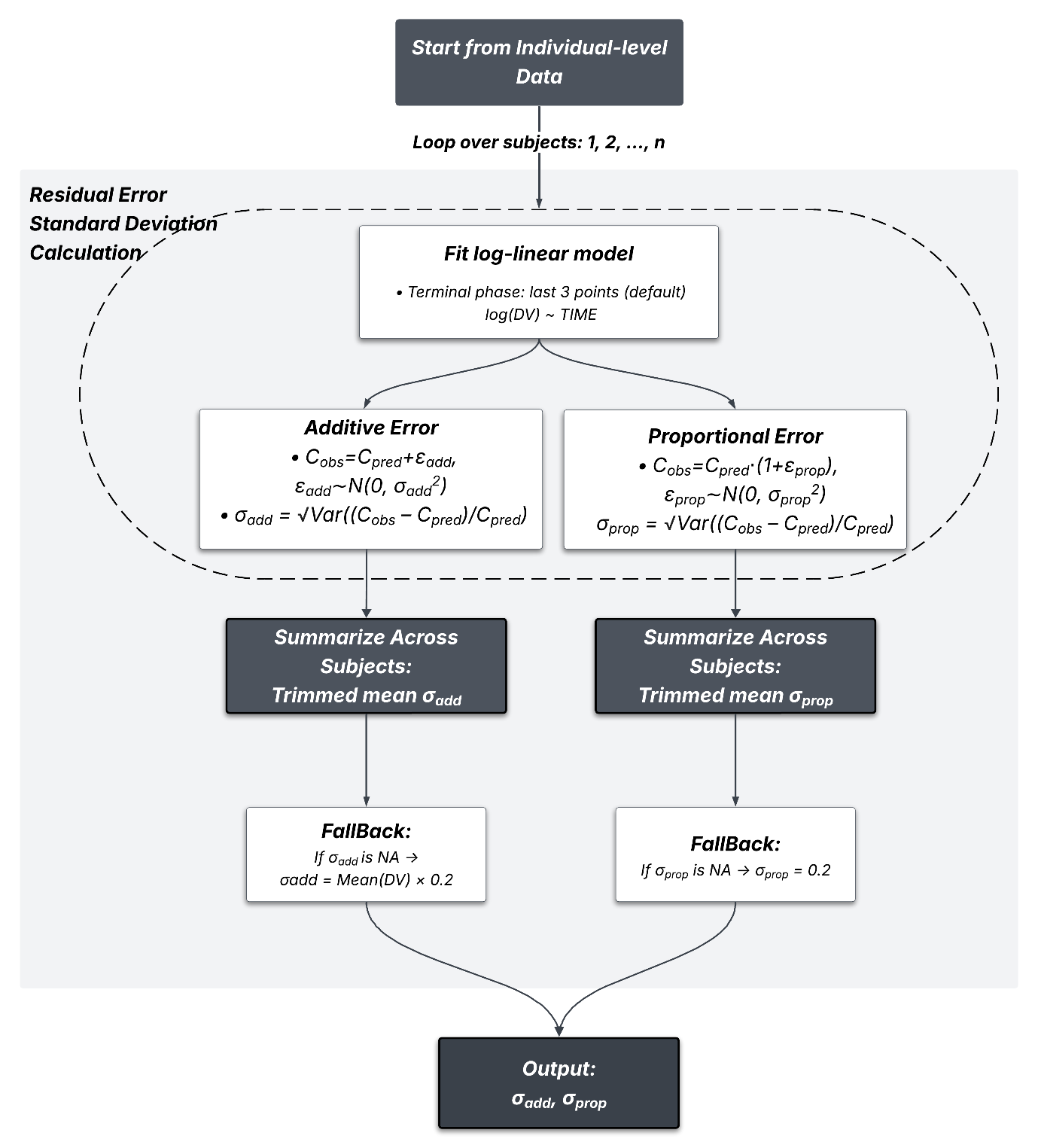


C_obs_: observed concentration, C_pred_: predicted concentration, σ_add_, σ_prop_: standard deviation of additive/proportional error, ε_add_, ε_prop_: error terms, DV: Dependent variable (concentration), log(DV): Log-transformed DV, Var: variance

**Reference**

1. Derendorf H, Schmidt S (2021) Chapter 11: Multiple-Dose Regimens. In: Rowland and Tozer’s Clinical Pharmacokinetics and Pharmacodynamics: Concepts and Applications, 5th ed. Wolters Kluwer, Philadelphia, PA

2. Lixoft (2024) Multiple doses and steady state. Monolix Documentation. https://monolixsuite.slp-software.com/monolix/2024R1/from-multiple-doses-to-steady-state. Accessed 11 June 2025

3. International Council for Harmonisation (2024) ICH M13A Guideline: Bioequivalence for Immediate-Release Solid Oral Dosage Forms (Step 5). https://www.ema.europa.eu/en/documents/scientific-guideline/ich-m13a-guideline-bioequivalence-immediaterelease-solid-oral-dosage-forms-step-5_en.pdf. Accessed 11 June 2025

4. Denhaerynck K, Dobbels F, Cleemput I, et al (2005) Prevalence, consequences, and determinants of nonadherence in adult renal transplant patients: a literature review. Transplant International 18:1121–1133. https://doi.org/10.1111/j.1432-2277.2005.00176.x

5. Rekk K, Arnet I, Dietrich F, et al (2024) Relationship between electronically monitored adherence to direct oral anticoagulants and ischemic or hemorrhagic events after an initial ischemic stroke—A case control study. PLOS ONE 19:e0301421. https://doi.org/10.1371/journal.pone.0301421

6. Jung H-Y, Jeon Y, Seong SJ, et al (2020) ICT-based adherence monitoring in kidney transplant recipients: a randomized controlled trial. BMC Medical Informatics and Decision Making 20:105. https://doi.org/10.1186/s12911-020-01146-6
