# Supplementary material for: An automated pipeline to generate initial estimates for population Pharmacokinetic base models
Source: J Pharmacokinet Pharmacodyn. 2025 Nov 6;52(6):60. doi: 10.1007/s10928-025-10000-z (PMC12592298; doi:10.1007/s10928-025-10000-z)
Supplement: Supplementary file 2 — (DOCX 7.29 MB) [file 10928_2025_10000_MOESM2_ESM.docx]

**An automated pipeline to generate initial estimates for population pharmacokinetic base models**

**(Supplementary Material 2)**

Zhonghui Huang^1^, Matthew Fidler^2^, Minshi Lan^1^, lek Leng Cheng^1,4^, Frank Kloprogge^3^, Joseph F Standing^1,4^

^1^Great Ormond Street Institute of Child Health, University College London, London, UK

^2^Novartis Pharmaceuticals Corporation, Fort Worth, Texas, USA

^3^Institute for Global Health, University College London, London, UK

^4^Great Ormond Street Hospital for Children, London, UK

**Corresponding author: Zhonghui Huang**

**Affiliation:** Great Ormond Street Institute of Child Health, University College London, London, UK

**E-mail:** [**zhonghui.huang.20@ucl.ac.uk**](mailto:zhonghui.huang.20@ucl.ac.uk)

**Supplementary Figures**

[**Supplementary Figure 1.** Plasma concentration plots for data collected from nlmixr2data package 2](#_Toc200708672)

[**Supplementary Figure 2.** Plasma concentration plots for ten real-life datasets collected from publications 3](#_Toc200708673)

[**Supplementary Figure 3.** Comparison of re-estimated clearance (up) and volume of distribution (bottom) across different strategies of setting initial estimates run by FOCEI 4](#_Toc200708674)

[**Supplementary Figure 4.** Model run time on 13 real-life datasets (SAEM) 5](#_Toc200708675)

[**Supplementary Figure 5**. Model run time on 13 real-life datasets (FOCEI) 6](#_Toc200708676)

[**Supplementary Figure 6.** Comparison of percentage deviations of initial parameter estimates from their true values based on three evaluation metrics. 7](#_Toc200708677)

[**Supplementary Figure 7.** Run time of designed pipeline for each test dataset 9](#_Toc200708678)

**Supplementary Tables**

[**Supplementary Table 1.** Summary of the study characteristics of 10 real-life datasets 10](#_Toc200708645)

[**Supplementary Table 2.** Initial and final pharmacokinetic parameter estimates across candidate methods in the pipeline and datasets, with their percentage deviations from true values 11](#_Toc200708646)

[**Supplementary Table 3.** Parameter re-estimates of 21 simulated datasets using different initial estimate strategies (SAEM) 14](#_Toc200708647)

[**Supplementary Table 4.** Parameter re-estimates of 21 simulated datasets using different initial estimate strategies (FOCEI) 18](#_Toc200708648)

[**Supplementary Table 5.** Statistics of final parameter estimates within 20% and 30% of original values across five initial estimate strategies in simulated datasets (SAEM) 22](#_Toc200708649)

[**Supplementary Table 6.** Statistics of final parameter estimates within 20% and 30% of original values across five initial estimate strategies in simulated datasets (FOCEI) 23](#_Toc200708650)

[**Supplementary Table 7.** Comparison of parameter estimation results using initial estimates set to 1 vs. pipeline recommendations for one- and two-compartment models (run by SAEM) 24](#_Toc200708651)

[**Supplementary Table 8** . Comparison between the OFV from using true values as initial estimates vs using pipeline values as initial estimate 29](#_Toc200708652)

[**Supplementary Table 9.** Initial estimates recommended by the pipeline for 13 real-life datasets 30](#_Toc200708653)

**Supplementary Figure 1.** Plasma concentration plots for data collected from nlmixr2data package

**

**

Open circles represent the observed data

**Supplementary Figure 2.** Plasma concentration plots for ten real-life datasets collected from publications

**

**

Open circles represent the observed data.

**Supplementary Figure 3.** Comparison of re-estimated clearance (up) and volume of distribution (bottom) across different strategies of setting initial estimates run by FOCEI


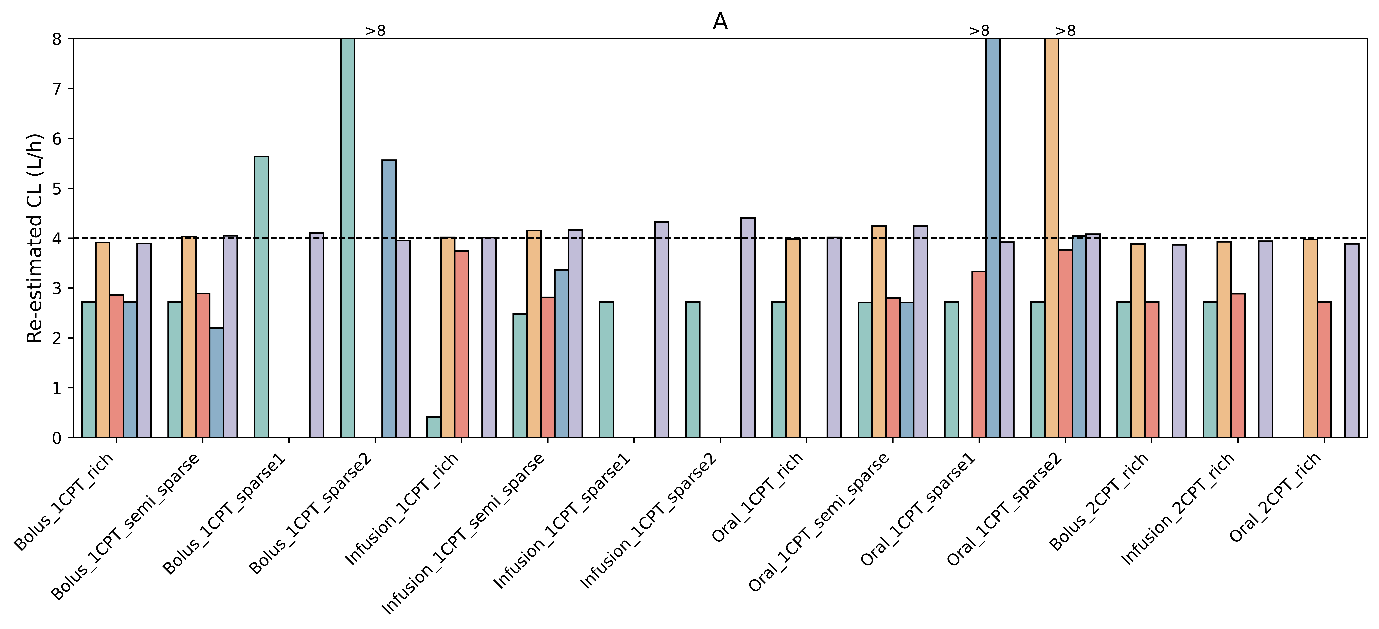

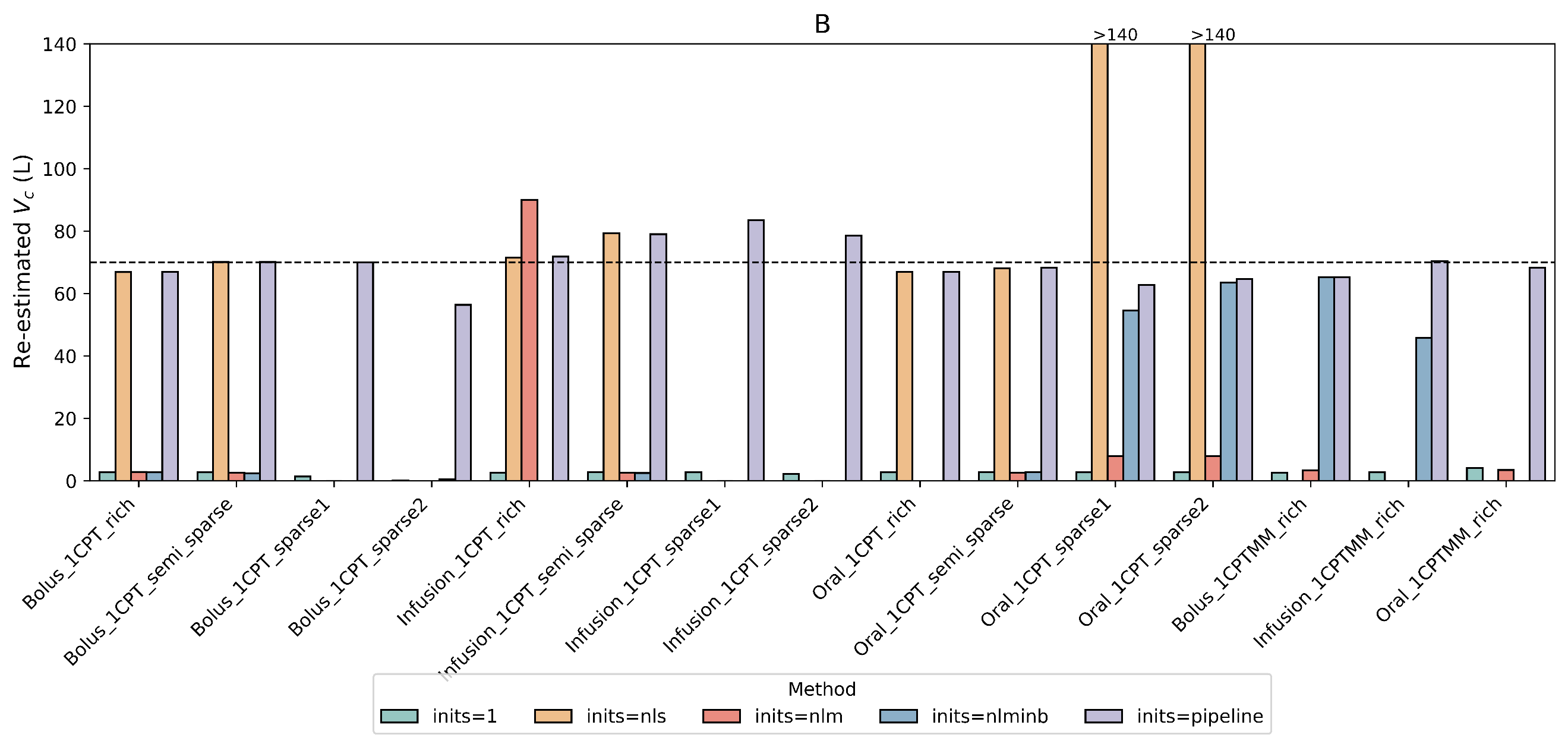


This figure contained re-estimation of clearance (A) and volume of distribution (B) in simulated datasets using five different initial estimate strategies, represented by distinct colors. inits = 1 sets all initial estimates to 1, while inits = nls, nits = nlm, and inits = nlminb used parameter estimates from respective algorithms as initial values. inits = pipeline referred to pipeline-specific recommendations. To address excessively large initial estimates, the y-axis was capped at 2-fold of the true values. Bars exceeding this limit are truncated at the 2-fold value and annotated with ">2-fold" to indicate their magnitude

**Supplementary Figure 4.** Model run time on 13 real-life datasets (SAEM)

**
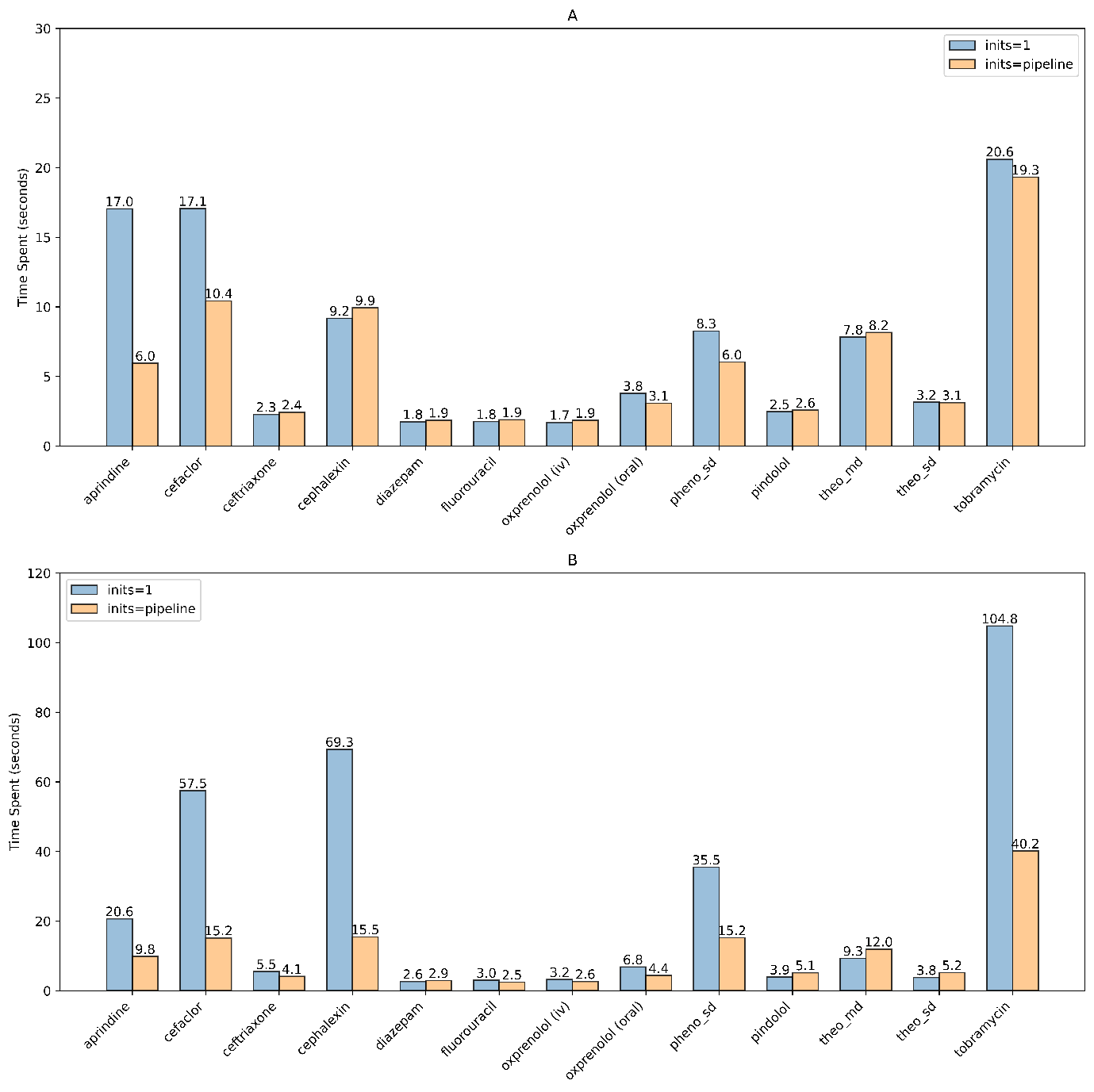
**

"inits = 1" sets all initial estimates to 1. "inits = pipeline" referred to pipeline-specific recommendations.

A. one-compartment model with first-order elimination (and first-order absorption for oral administration). B. two-compartment model with first-order elimination (and first-order absorption for oral administration).

**Supplementary Figure 5**. Model run time on 13 real-life datasets (FOCEI)

**
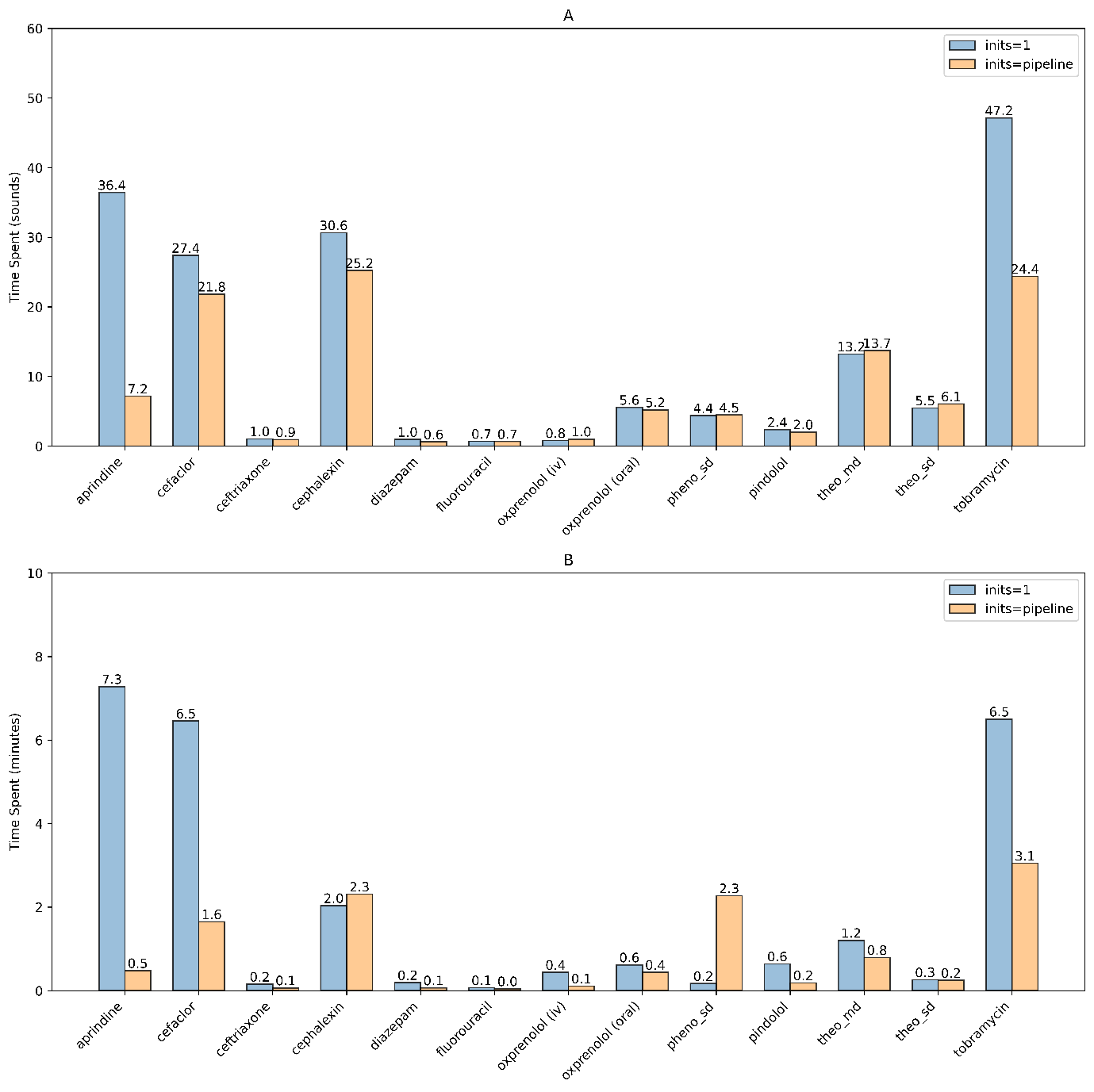
**

"inits = 1" sets all initial estimates to 1. "inits = pipeline" referred to pipeline-specific recommendations.

A. one-compartment model with first-order elimination (and first-order absorption for oral administration). B. two-compartment model with first-order elimination (and first-order absorption for oral administration).

**Supplementary Figure 6.** Comparison of percentage deviations of initial parameter estimates from their true values based on three evaluation metrics.


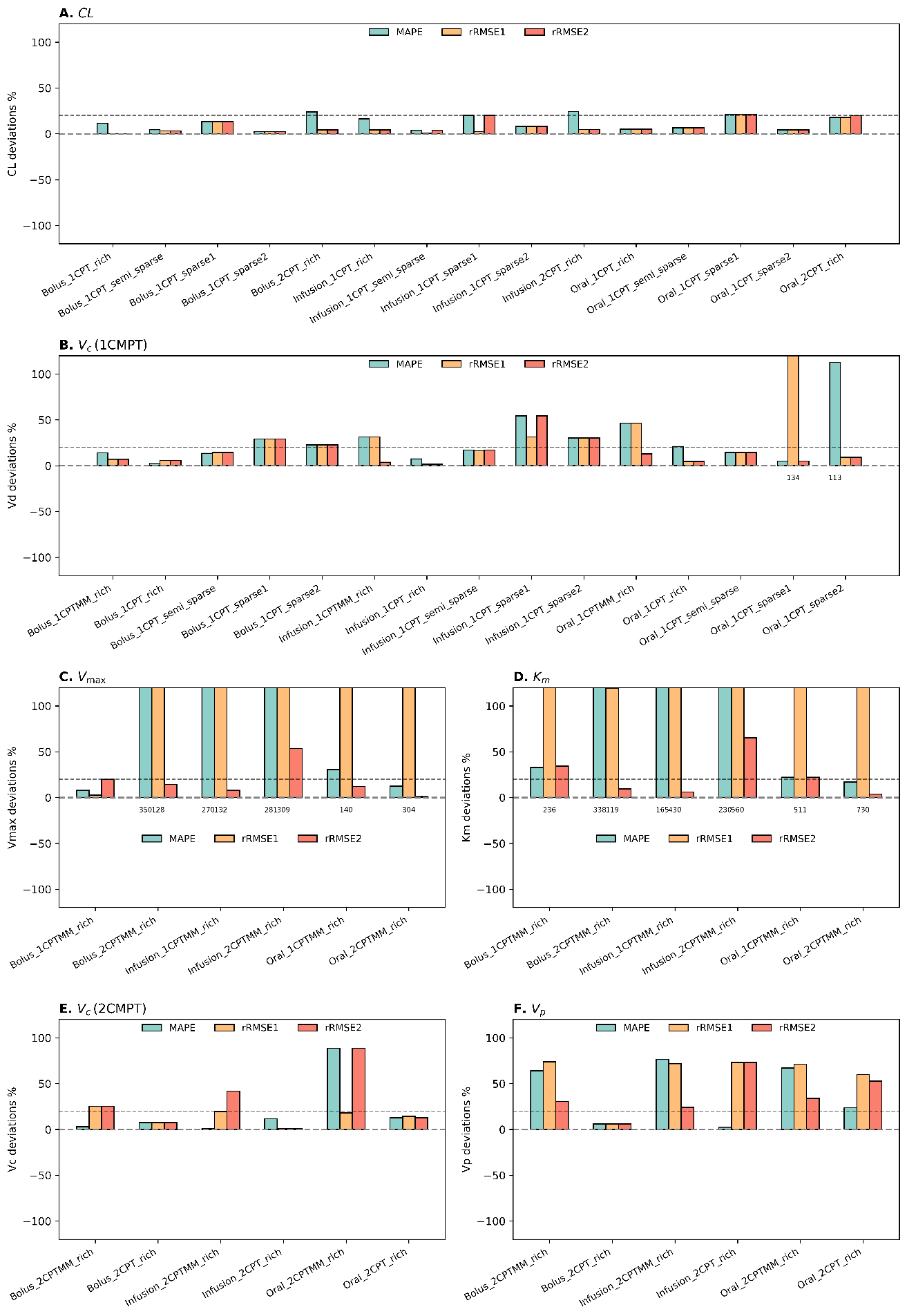


This figure compares the percentage deviations of initial estimates for pharmacokinetic parameters (CL, V_d_, V_max_, K_m_, V_c_ (1CMPT), V_c_ (2CMPT) and V_p_) from their true values used in the simulation. The initial estimates were obtained from the pipeline using one of three evaluation metrics: MAPE (blue-green), rRMSE1 (orange), and rRMSE2 (red). A black dashed lines at 20% denote reference deviation thresholds. Bars exceeding ±100% are annotated. This analysis highlights how the choice of metric affects the selection of initial parameter values how close these initial estimates are to the true values. MAPE: mean absolute percentage error, calculated as the absolute difference divided by the observed value at each time point. rRMSE1: relative root mean square error, calculated as the root mean square error (RMSE) normalized by the mean of observed values. rRMSE2: relative RMSE, where each squared error was scaled by the pointwise average of predicted and observed values, default method set in the pipeline. CL: clearance, V_c_ (1CMPT): the central volume of distribution in a one-compartment model, V_max_: the maximum elimination rate, K_m_: the Michaelis-Menten constant, V_c_ (2CMPT): the central volume in a two-compartment model, V_p_: the peripheral volume of distribution.

**Supplementary Figure 7.** Run time of designed pipeline for each test dataset


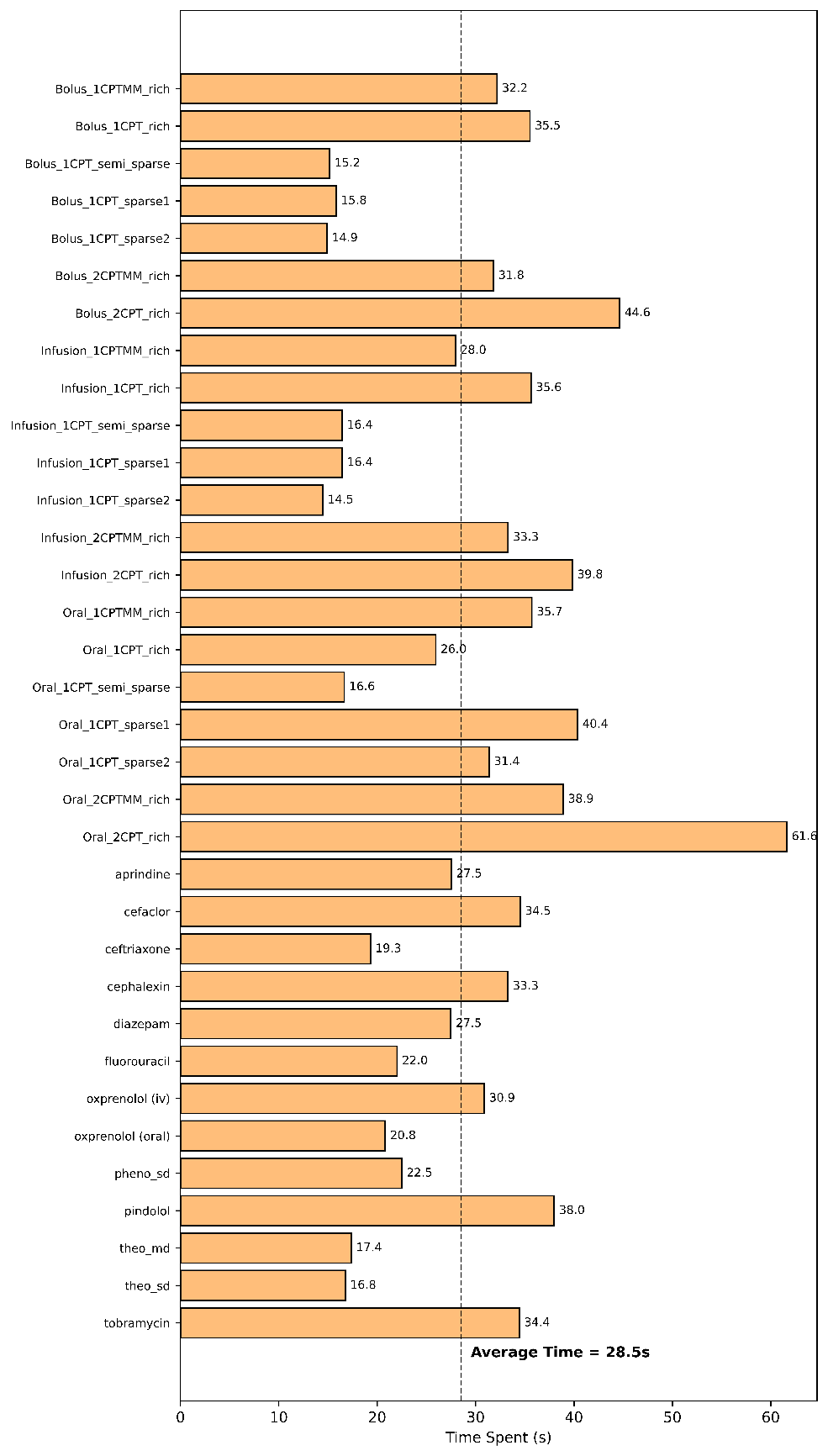


**Supplementary Table 1.** Summary of the study characteristics of 10 real-life datasets

| Dataset name | No. of subjects (samples) | Population | Route | Dosing information | Sampling information |
| --- | --- | --- | --- | --- | --- |
| Aprindine [1] | 21 (251)^a^ | Patients | Oral | Multiple doses (200mg at 0h, 100mg at 2h, 100mg at every 12h ) | 0.5, 1, 1.5, 2, 2.5, 3.0, 6.0, 12 h after the initial dose; 0, 1.0, 3.0, 6.0, 12.0, 24.0, 48.0 h, after the last dose (at 60 hours) |
| Cefaclor [2] | 20 (199) | Healthy volunteers | Oral | 250 mg at every 6 hours | 0.5, 0.75, 1.0, 1.5, 2.0, 3.0, 4.0 after initial dose; 72.5, 73.0, 73.5, 74.0, 75.0, 76.0 on day 4 |
| Ceftriaxone [3] | 20 (64) | Neonates | Infusion | 50 mg/kg or 100 mg/kg (infused over 5 minutes) | 1, 12, 24 h (after termination of the infusion)^b^ |
| Cephalexin [2] | 20 (264) | Healthy volunteers | Oral | 250 mg at every 6 hours | 0.5, 0.75, 1.0, 1.5, 2.0, 3.0, 4.0 after initial dose; 72.5, 73.0, 73.5, 74.0, 75.0, 76.0 h on day 4 |
| Diazepam [4, 5] | 14 (149) | Healthy volunteers | Injection | Single injection (10 or 20 mg) | Intensive sampling after dosing (11 samples or 19 samples for a subject) |
| Fluorouracil [6, 7] | 13 (66) | Patients | Injection | Single injection (varied doses) | 5, 10 15, 20, 30, 45, 60, 90, 120 min after initial dose or 5, 10, 20, 30, 60 min after initial dose |
| Oxprenolol (iv) [8] | 6 (102) | Healthy volunteers | Infusion | Single infusion (10 or 20 mg over a 10-min infusion) | 10 (min), 25 (min), 40 (min), 1, 2, 3, 4, 6, 8h ( from the time the  infusion was begun) |
| Oxprenolol (oral) [8] | 6 (203) | Healthy volunteers | Oral | Single dose (20, 40, 80, 160mg) | 0.25, 0.5 1, 2, 4, 6, 8 h |
| Pindolol (iv) [9] | 12 (104) | Healthy volunteers | Oral | Single dose (5mg) | 0.5, 1.0, 2.0, 3.0, 4.0, 6.0, 8.0, 12.0, 24.0 h |
| Tobramycin [10] | 97 (322) | Patients | Bolus/short Infusion | Multiple doses (varied doses) | Sparse sampling (varied time) |

^a^ The number of subjects (samples) is the actual count in the analysis dataset, excluding those with BQLs.

**Supplementary Table 2.** Initial and final pharmacokinetic parameter estimates across candidate methods in the pipeline and datasets, with their percentage deviations from true values

| Dataset | Adaptive single-point method | Graphic methods | Naïve pooled NCA (first dose) | Naïve pooled NCA (multiple dose) | Naïve pooled NCA (all) |
| --- | --- | --- | --- | --- | --- |
| Bolus_1CPT (rich) | CL_init_ = 2.97 (vs final: 3.90 (23.8%), vs true 4.0 (25.7%)), Vd_init_ = 67.60 (vs final: 66.83 (1.2%), vs true 70 (3.4%)) | CL_init_ = 4.20 (vs final: 3.90 (7.7%), vs true 4.0 (5.0%)), Vd_init_ = 66.60 (vs final: 66.83 (0.3%), vs true 70 (4.9%)) | CL_init_ = 4.17 (vs final: 3.90 (6.9%), vs true 4.0 (4.2%)), Vd_init_ = 66.20 (vs final: 66.83 (0.9%), vs true 70 (5.4%)) | **CL_init_ = 4.00 (vs final: 3.90 (2.6%), vs true 4.0 (0.0%)), Vd_init_ = 66.00 (vs final: 66.83 (1.2%), vs true 70 (5.7%))** | CL_init_ = 4.46 (vs final: 3.90 (14.4%), vs true 4.0 (11.5%)), Vd_init_ = 71.90 (vs final: 66.84 (7.6%), vs true 70 (2.7%)) |
| Bolus_1CPT (semi-sparse) | CL_init_ = 4.18 (vs final: 3.94 (6.0%), vs true 4.0 (4.5%)), Vd_init_ = 60.60 (vs final: 71.90 (15.7%), vs true 70 (13.4%)) |  |  | **CL_init_ = 4.13 (vs final: 3.94 (4.8%), vs true 4.0 (3.2%)), Vd_init_ = 59.90 (vs final: 72.31 (17.2%), vs true 70 (14.4%))** |  |
| Bolus_1CPT (sparse1) | CL_init_ = 3.80 (vs final: 4.02 (5.5%), vs true 4.0 (5.0%)), Vd_init_ = 75.70 (vs final: 69.87 (8.3%), vs true 70 (8.1%)) |  |  | **CL_init_ = 4.54 (vs final: 4.03 (12.8%), vs true 4.0 (13.5%)), Vd_init_ = 90.40 (vs final: 70.36 (28.5%), vs true 70 (29.1%))** |  |
| Bolus_1CPT (sparse2) |  | **CL_init_ = 3.90 (vs final: 3.84 (1.7%), vs true 4.0 (2.5%)), Vd_init_ = 54.20 (vs final: 55.60 (2.5%), vs true 70 (22.6%))** | **CL_init_ = 3.90 (vs final: 3.84 (1.7%), vs true 4.0 (2.5%)), Vd_init_ = 54.20 (vs final: 55.60 (2.5%), vs true 70 (22.6%))** |  |  |
| Infusion_1CPT (rich) | CL_init_ = 3.16 (vs final: 4.00 (21.1%), vs true 4.0 (21.0%)), Vd_init_ = 72.70 (vs final: 71.61 (1.5%), vs true 70 (3.9%)) | CL_init_ = 4.22 (vs final: 4.00 (5.4%), vs true 4.0 (5.5%)), Vd_init_ = 68.10 (vs final: 71.60 (4.9%), vs true 70 (2.7%)) | CL_init_ = 4.33 (vs final: 4.00 (8.1%), vs true 4.0 (8.3%)), Vd_init_ = 69.90 (vs final: 71.64 (2.4%), vs true 70 (0.1%)) | **CL_init_ = 4.17 (vs final: 4.00 (4.1%), vs true 4.0 (4.2%)), Vd_init_ = 68.80 (vs final: 71.62 (3.9%), vs true 70 (1.7%))** | CL_init_ = 4.65 (vs final: 4.00 (16.1%), vs true 4.0 (16.3%)), Vd_init_ = 75.20 (vs final: 71.63 (5.0%), vs true 70 (7.4%)) |
| Infusion_1CPT (semi-sparse) | CL_init_ = 4.04 (vs final: 4.02 (0.5%), vs true 4.0 (1.0%)), Vd_init_ = 81.40 (vs final: 81.23 (0.2%), vs true 70 (16.3%)) |  |  | **CL_init_ = 4.15 (vs final: 4.02 (3.3%), vs true 4.0 (3.8%)), Vd_init_ = 81.90 (vs final: 81.21 (0.9%), vs true 70 (17.0%))** |  |
| Infusion_1CPT (sparse1) | CL_init_ = 4.10 (vs final: 4.15 (1.2%), vs true 4.0 (2.5%)), Vd_init_ = 92.00 (vs final: 79.06 (16.4%), vs true 70 (31.4%)) |  |  | **CL_init_ = 4.80 (vs final: 4.17 (15.2%), vs true 4.0 (20.0%)), Vd_init_ = 108.00 (vs final: 80.04 (34.9%), vs true 70 (54.3%))** |  |
| Infusion_1CPT (sparse2) |  | **CL_init_ = 4.32 (vs final: 3.92 (10.3%), vs true 4.0 (8.0%)), Vd_init_ = 91.30 (vs final: 72.63 (25.7%), vs true 70 (30.4%))** | **CL_init_ = 4.32 (vs final: 3.92 (10.3%), vs true 4.0 (8.0%)), Vd_init_ = 91.30 (vs final: 72.63 (25.7%), vs true 70 (30.4%))** |  |  |
| Oral_1CPT  (rich) | CL_init_ = 3.29 (vs final: 4.01 (17.9%), vs true 4.0 (17.8%)), Vd_init_ = 55.30 (vs final: 66.84 (17.3%), vs true 70 (21.0%)), Ka_init_ = 0.64 (vs final: 1.00 (35.9%), vs true 1 (35.6%)) | **CL_init_ = 4.20 (vs final: 4.01 (4.8%), vs true 4.0 (5.0%)), Vd_init_ = 66.70 (vs final: 66.86 (0.2%), vs true 70 (4.7%)), Ka_init_ = 0.89 (vs final: 1.00 (11.6%), vs true 1 (11.2%))** | CL_init_ = 4.20 (vs final: 4.01 (4.8%), vs true 4.0 (5.0%)), Vd_init_ = 67.10 (vs final: 66.87 (0.3%), vs true 70 (4.1%)), Ka_init_ = 0.82 (vs final: 1.00 (18.2%), vs true 1 (17.8%)) | CL_init_ = 4.12 (vs true 4.0 (3.1%)), Vd_init_ = 70.58 (vs true 70 (0.8%)) | CL_init_ = 4.59 (vs true 4.0 (14.8%)), Vd_init_ = 77.17 (vs true 70 (10.2%)) |
| Oral_1CPT (semi-sparse) | **CL_init_ = 4.27 (vs final: 4.08 (4.7%), vs true 4.0 (6.7%)), Vd_init_ = 80.00 (vs final: 70.77 (13.0%), vs true 70 (14.3%)), Ka_init_ = 0.40 (vs final: 1.25 (68.4%), vs true 1 (60.5%))** |  |  | CL_init_ = 4.11 (vs true 4.0 (2.7%)), Vd_init_ = 76.95 (vs true 70 (9.9%)) |  |
| Oral_1CPT (sparse1) | **CL_init_ = 4.85 (vs final: 4.13 (17.3%), vs true 4.0 (21.2%)), Vd_init_ = 73.50 (vs final: 79.13 (7.1%), vs true 70 (5.0%)), Ka_init_ = 1.52 (vs final: 1.24 (22.4%), vs true 1 (52.0%))** |  |  | CL_init_ = 5.24 (vs true 4.0 (30.9%)), Vd_init_ = 163.95 (vs true 70 (134.2%)) |  |
| Oral_1CPT (sparse2) | CL_init_ = 8.16 (vs final: 3.93 (107.5%), vs true 4.0 (104.0%)), Vd_init_ = 149.00 (vs final: 69.82 (113.4%), vs true 70 (112.9%)), Ka_init_ = 1.96 (vs final: 1.04 (88.9%), vs true 1 (96.0%)) | **CL_init_ = 4.17 (vs final: 3.90 (6.8%), vs true 4.0 (4.2%)), Vd_init_ = 76.30 (vs final: 64.53 (18.2%), vs true 70 (9.0%)), Ka_init_ = 1.12 (vs final: 0.93 (19.9%), vs true 1 (12.0%))** | CL_init_ = 2.69 (vs true 4.0 (32.8%)), Vd_init_ = 184.06 (vs true 70 (162.9%)) |  |  |

Note: Each cell presents the initial pharmacokinetic (PK) parameter estimates (e.g., CL_init_ = : initial parameter estimates of clearance; Vd_init_ = Initial parameter estimates of volume of distribution; Ka_init_ = initial parameter estimates of absorption rate constant), followed by two comparisons in parentheses: (1) the percentage difference between the initial estimate and the final model estimate, and (2) the percentage difference between the initial estimate and the known true value used in simulation. The bold values represent the methods selected by the pipeline based on the lowest rRMSE after evaluation.

**Supplementary Table 3.** Parameter re-estimates of 21 simulated datasets using different initial estimate strategies (SAEM)

| Run cases | k_a_ | CL | V_c_ | V_p_ | Q | V_max_ | K_m_ |
| --- | --- | --- | --- | --- | --- | --- | --- |
| *Inits=1 ^a^* |  |  |  |  |  |  |  |
| Bolus_1CPT_rich |  | 3.9 [3.0] ^b^ | 66.8 [5.0] |  |  |  |  |
| Bolus_1CPT_semi_sparse |  | 3.94 [2.0] | 71.7 [2.0] |  |  |  |  |
| Bolus_1CPT_sparse1 |  | 1.42 [64.0] | 10.9 [84.0] |  |  |  |  |
| Bolus_1CPT_sparse2 |  | 1.39 [65.0] | 9.26 [87.0] |  |  |  |  |
| Bolus_1CPTMM_rich |  |  | 65.4 [7.0] |  |  | 994 [1.0] | 254 [2.0] |
| Bolus_2CPT_rich |  | 3.8 [5.0] | 53.9 [23.0] | 49.8 [24.0] | 3.99 [0.0] |  |  |
| Bolus_2CPTMM_rich |  |  | 74 [6.0] | 358 [795.0] | 3.69 [8.0] | 31.1 [97.0] | 2.55 [99.0] |
| Infusion_1CPT_rich |  | 4 [0.0] | 71.6 [2.0] |  |  |  |  |
| Infusion_1CPT_semi_sparse |  | 4.02 [0.0] | 80.9 [16.0] |  |  |  |  |
| Infusion_1CPT_sparse1 |  | 1.48 [63.0] | 11.6 [83.0] |  |  |  |  |
| Infusion_1CPT_sparse2 |  | 1.38 [66.0] | 9.03 [87.0] |  |  |  |  |
| Infusion_1CPTMM_rich |  |  | 70.4 [1.0] |  |  | 997 [0.0] | 242 [3.0] |
| Infusion_2CPT_rich |  | 3.92 [2.0] | 67.9 [3.0] | 50.2 [26.0] | 3.92 [2.0] |  |  |
| Infusion_2CPTMM_rich |  |  | 72.7 [4.0] | 257 [542.0] | 3.7 [7.0] | 45.9 [95.0] | 2.52 [99.0] |
| Oral_1CPT_rich | 0.0595 [94.0] | 4.96e+03 [1.24e+05] | 4.75e+03 [6.7e+03] |  |  |  |  |
| Oral_1CPT_semi_sparse | 4.51 [351.0] | 3.94 [2.0] | 72.2 [3.0] |  |  |  |  |
| Oral_1CPT_sparse1 | 0.512 [49.0] | 3.19 [20.0] | 38.7 [45.0] |  |  |  |  |
| Oral_1CPT_sparse2 | 0.39 [61.0] | 3.32 [17.0] | 30 [57.0] |  |  |  |  |
| Oral_1CPTMM_rich | 1 [0.0] |  | 68.8 [2.0] |  |  | 979 [2.0] | 239 [4.0] |
| Oral_2CPT_rich | 0.833 [17.0] | 3.85 [4.0] | 58.6 [16.0] | 51.2 [28.0] | 5.03 [26.0] |  |  |
| Oral_2CPTMM_rich | 1.12 [12.0] |  | 76.6 [9.0] | 380 [850.0] | 3.82 [5.0] | 4.24 [100.0] | 2.42 [99.0] |
|  |  |  |  |  |  |  |  |
| *Inits=nls* |  |  |  |  |  |  |  |
| Bolus_1CPT_rich |  | 3.9 [3.0] | 66.8 [5.0] |  |  |  |  |
| Bolus_1CPT_semi_sparse |  | 3.94 [2.0] | 71.9 [3.0] |  |  |  |  |
| Bolus_2CPT_rich |  | 3.87 [3.0] | 65.7 [6.0] | 49.5 [24.0] | 4.04 [1.0] |  |  |
| Infusion_1CPT_rich |  | 4 [0.0] | 71.6 [2.0] |  |  |  |  |
| Infusion_1CPT_semi_sparse |  | 4.02 [0.0] | 81.2 [16.0] |  |  |  |  |
| Infusion_2CPT_rich |  | 3.92 [2.0] | 68 [3.0] | 50.1 [25.0] | 3.9 [3.0] |  |  |
| Oral_1CPT_rich | 1.01 [1.0] | 4.01 [0.0] | 66.9 [4.0] |  |  |  |  |
| Oral_1CPT_semi_sparse | 2.19 [119.0] | 4 [0.0] | 72.2 [3.0] |  |  |  |  |
| Oral_1CPT_sparse1 | 2.72 [172.0] | 2.78e-74 [100.0] | 1.26e+22 [1.8e+22] |  |  |  |  |
| Oral_1CPT_sparse2 | 2.72 [172.0] | 2.59e-74 [100.0] | 1.14e+22 [1.63e+22] |  |  |  |  |
| Oral_2CPT_rich | 1.67 [67.0] | 3.77 [6.0] | 105 [50.0] | 1e+11 [2.5e+11] | 4.06e-11 [100.0] |  |  |
|  |  |  |  |  |  |  |  |
| *Inits=nlm* |  |  |  |  |  |  |  |
| Bolus_1CPT_rich |  | 3.9 [3.0] | 66.8 [5.0] |  |  |  |  |
| Bolus_1CPT_semi_sparse |  | 3.94 [2.0] | 72 [3.0] |  |  |  |  |
| Bolus_1CPT_sparse1 |  | 0.00227 [100.0] | 0 [100.0] |  |  |  |  |
| Bolus_1CPTMM_rich |  |  | 65.4 [7.0] |  |  | 999 [0.0] | 257 [3.0] |
| Bolus_2CPT_rich |  | 3.8 [5.0] | 53.9 [23.0] | 49.8 [24.0] | 3.99 [0.0] |  |  |
| Infusion_1CPT_rich |  | 4 [0.0] | 71.6 [2.0] |  |  |  |  |
| Infusion_1CPT_semi_sparse |  | 4.02 [0.0] | 81.1 [16.0] |  |  |  |  |
| Infusion_1CPT_sparse1 |  | 3.58e-07 [100.0] | 5.09e-11 [100.0] |  |  |  |  |
| Infusion_1CPT_sparse2 |  | 7.16e-07 [100.0] | 0 [100.0] |  |  |  |  |
| Infusion_1CPTMM_rich |  |  | 70.4 [1.0] |  |  | 1e+03 [0.0] | 246 [2.0] |
| Infusion_2CPT_rich |  | 3.92 [2.0] | 68 [3.0] | 50.2 [26.0] | 3.9 [3.0] |  |  |
| Oral_1CPT_semi_sparse | 3.98 [298.0] | 3.95 [1.0] | 72.1 [3.0] |  |  |  |  |
| Oral_1CPT_sparse1 | 0.593 [41.0] | 3.36 [16.0] | 44.8 [36.0] |  |  |  |  |
| Oral_1CPT_sparse2 | 0.416 [58.0] | 3.38 [16.0] | 31.9 [54.0] |  |  |  |  |
| Oral_1CPTMM_rich | 0.999 [0.0] |  | 68.8 [2.0] |  |  | 987 [1.0] | 245 [2.0] |
| Oral_2CPT_rich | 1.42 [42.0] | 4.32 [8.0] | 3.09 [96.0] | 83.6 [109.0] | 8.64 [116.0] |  |  |
| Oral_2CPTMM_rich | 0.895 [10.0] |  | 66 [6.0] | 316 [690.0] | 3.59 [10.0] | 43.4 [96.0] | 3.53 [99.0] |
|  |  |  |  |  |  |  |  |
| *Inits=nlminb* |  |  |  |  |  |  |  |
| Bolus_1CPT_rich |  | 3.9 [3.0] | 66.8 [5.0] |  |  |  |  |
| Bolus_1CPT_semi_sparse |  | 3.95 [1.0] | 71.3 [2.0] |  |  |  |  |
| Bolus_1CPT_sparse2 |  | 1.41 [65.0] | 9.42 [87.0] |  |  |  |  |
| Bolus_1CPTMM_rich |  |  | 65.3 [7.0] |  |  | 1e+03 [0.0] | 258 [3.0] |
| Infusion_1CPT_rich |  | 5.95e-12 [100.0] | 274 [291.0] |  |  |  |  |
| Infusion_1CPT_semi_sparse |  | 4.02 [0.0] | 81.2 [16.0] |  |  |  |  |
| Infusion_1CPTMM_rich |  |  | 9.53e+04 [1.36e+05] |  |  | 3.34e+07 [3.34e+06] | 0.00112 [100.0] |
| Infusion_2CPT_rich |  | 7.21e-12 [100.0] | 88.3 [26.0] | 1.78e+05 [4.45e+05] | 3.79 [5.0] |  |  |
| Oral_1CPT_rich | 5.66 [466.0] | 1.51e-09 [100.0] | 320 [357.0] |  |  |  |  |
| Oral_1CPT_semi_sparse | 4.51 [351.0] | 3.94 [2.0] | 72.2 [3.0] |  |  |  |  |
| Oral_1CPT_sparse1 | 2.72 [172.0] | 9.69e+15 [2.42e+17] | 54.6 [22.0] |  |  |  |  |
| Oral_1CPT_sparse2 | 1.01 [1.0] | 3.93 [2.0] | 68.5 [2.0] |  |  |  |  |
| Oral_2CPTMM_rich | 1.7 [70.0] |  | 102 [46.0] | 1.38e+29 [3.45e+29] | 0.735 [82.0] | 230 [77.0] | 23.9 [90.0] |
|  |  |  |  |  |  |  |  |
| *Inits=pipeline* |  |  |  |  |  |  |  |
| Bolus_1CPT_rich |  | 3.9 [2.5] | 66.8 [4.5] |  |  |  |  |
| Bolus_1CPT_semi_sparse |  | 3.94 [1.5] | 72.3 [3.3] |  |  |  |  |
| Bolus_1CPT_sparse1 |  | 4.03 [0.7] | 70.4 [0.5] |  |  |  |  |
| Bolus_1CPT_sparse2 |  | 3.84 [4.1] | 55.6 [20.6] |  |  |  |  |
| Bolus_1CPTMM_rich |  |  | 65.3 [6.7] |  |  | 998 [0.2] | 256 [2.6] |
| Bolus_2CPT_rich |  | 3.87 [3.3] | 65.7 [6.1] | 49.5 [23.8] | 4.04 [0.9] |  |  |
| Bolus_2CPTMM_rich |  |  | 70.4 [0.5] | 51.2 [28.1] | 3.98 [0.6] | 986 [1.4] | 240 [4.0] |
| Infusion_1CPT_rich |  | 4 [0.1] | 71.6 [2.3] |  |  |  |  |
| Infusion_1CPT_semi_sparse |  | 4.02 [0.4] | 81.2 [16.0] |  |  |  |  |
| Infusion_1CPT_sparse1 |  | 4.14 [3.5] | 79.8 [14.0] |  |  |  |  |
| Infusion_1CPT_sparse2 |  | 3.92 [2.1] | 72.6 [3.8] |  |  |  |  |
| Infusion_1CPTMM_rich |  |  | 70.3 [0.4] |  |  | 1.01e+03 [0.8] | 250 [0.2] |
| Infusion_2CPT_rich |  | 3.92 [1.9] | 68 [2.9] | 50.2 [25.5] | 3.91 [2.2] |  |  |
| Infusion_2CPTMM_rich |  |  | 70.3 [0.4] | 50.3 [25.7] | 3.92 [2.1] | 1.02e+03 [1.6] | 249 [0.3] |
| Oral_1CPT_rich | 1 [0.4] | 4.01 [0.2] | 66.9 [4.5] |  |  |  |  |
| Oral_1CPT_semi_sparse | 1.25 [24.9] | 4.08 [1.9] | 70.8 [1.1] |  |  |  |  |
| Oral_1CPT_sparse1 | 1.24 [24.2] | 4.13 [3.3] | 79.1 [13.0] |  |  |  |  |
| Oral_1CPT_sparse2 | 0.934 [6.6] | 3.9 [2.4] | 64.5 [7.8] |  |  |  |  |
| Oral_1CPTMM_rich | 0.992 [0.8] |  | 68.3 [2.5] |  |  | 1e+03 [0.1] | 251 [0.3] |
| Oral_2CPT_rich | 0.919 [8.1] | 3.87 [3.2] | 67.3 [3.9] | 47.5 [18.8] | 4.32 [7.9] |  |  |
| Oral_2CPTMM_rich | 0.991 [0.9] |  | 70.1 [0.2] | 50.3 [25.7] | 3.89 [2.7] | 984 [1.6] | 258 [3.1] |

Cases that failed to get parameter estimates were not listed in the table.

^a^ "*inits = 1*" sets all initial estimates to 1, while "*inits = nls*," "*inits = nlm*," and "*inits = nlminb*" used parameter estimates from respective algorithms as initial values. "*inits = pipeline*" referred to pipeline-specific recommendations.

^b^ Values represent the estimated value, along with its relative error compared to original values in the simulated dataset. The original values using for generating simulated dataset are as follows: k_a_ = 1 h^-1^, CL = 4 L/h, V_c_ = 70 L, V_p_ = 40 L, Q = 4 L/h, V_max_ = 1000 mg/h, and K_m_ = 250 mg/L.

**Supplementary Table 4.** Parameter re-estimates of 21 simulated datasets using different initial estimate strategies (FOCEI)

| Dataset | Ka | CL | V_c_ | V_p_ | Q | V_max_ | K_m_ |
| --- | --- | --- | --- | --- | --- | --- | --- |
| *Inits=1* |  |  |  |  |  |  |  |
| Bolus_1CPT_rich |  | 2.72 [32.0] | 2.72 [96.0] |  |  |  |  |
| Bolus_1CPT_semi_sparse |  | 2.72 [32.0] | 2.72 [96.0] |  |  |  |  |
| Bolus_1CPT_sparse1 |  | 5.64 [41.0] | 1.37 [98.0] |  |  |  |  |
| Bolus_1CPT_sparse2 |  | 103 [2475.0] | 0.0879 [100.0] |  |  |  |  |
| Bolus_1CPTMM_rich |  |  | 2.56 [96.0] |  |  | 2.72 [100.0] | 2.73 [99.0] |
| Bolus_2CPT_rich |  | 2.72 [32.0] | 2.72 [96.0] | 2.72 [93.0] | 2.72 [32.0] |  |  |
| Bolus_2CPTMM_rich |  |  | 72.9 [4.0] | 479 [1098.0] | 3.54 [12.0] | 1.17 [100.0] | 14.5 [94.0] |
| Infusion_1CPT_rich |  | 0.41 [90.0] | 2.6 [96.0] |  |  |  |  |
| Infusion_1CPT_semi_sparse |  | 2.48 [38.0] | 2.74 [96.0] |  |  |  |  |
| Infusion_1CPT_sparse1 |  | 2.72 [32.0] | 2.72 [96.0] |  |  |  |  |
| Infusion_1CPT_sparse2 |  | 2.72 [32.0] | 2.23 [97.0] |  |  |  |  |
| Infusion_1CPTMM_rich |  |  | 2.72 [96.0] |  |  | 2.72 [100.0] | 2.72 [99.0] |
| Infusion_2CPT_rich |  | 2.72 [32.0] | 2.72 [96.0] | 2.72 [93.0] | 2.72 [32.0] |  |  |
| Infusion_2CPTMM_rich |  |  | 70.6 [1.0] | 50.7 [27.0] | 3.85 [4.0] | 954 [5.0] | 213 [15.0] |
| Oral_1CPT_rich | 2.72 [172.0] | 2.72 [32.0] | 2.72 [96.0] |  |  |  |  |
| Oral_1CPT_semi_sparse | 2.27 [127.0] | 2.71 [32.0] | 2.75 [96.0] |  |  |  |  |
| Oral_1CPT_sparse1 | 1 [0.0] | 2.72 [32.0] | 2.72 [96.0] |  |  |  |  |
| Oral_1CPT_sparse2 | 1 [0.0] | 2.72 [32.0] | 2.72 [96.0] |  |  |  |  |
| Oral_1CPTMM_rich | 3.06 [206.0] |  | 4.11 [94.0] |  |  | 2.95 [100.0] | 6.64 [97.0] |
| Oral_2CPT_rich | 2.72 [172.0] | 2.72 [32.0] | 2.72 [96.0] | 2.72 [93.0] | 2.72 [32.0] |  |  |
|  |  |  |  |  |  |  |  |
| *Inits=nls* |  |  |  |  |  |  |  |
| Bolus_1CPT_rich |  | 3.91 [2.0] | 66.9 [4.0] |  |  |  |  |
| Bolus_1CPT_semi_sparse |  | 4.03 [1.0] | 70.1 [0.0] |  |  |  |  |
| Bolus_2CPT_rich |  | 3.88 [3.0] | 66.1 [6.0] | 49.2 [23.0] | 4.01 [0.0] |  |  |
| Infusion_1CPT_rich |  | 4.02 [0.0] | 71.5 [2.0] |  |  |  |  |
| Infusion_1CPT_semi_sparse |  | 4.15 [4.0] | 79.4 [13.0] |  |  |  |  |
| Infusion_2CPT_rich |  | 3.93 [2.0] | 68.1 [3.0] | 49.5 [24.0] | 3.88 [3.0] |  |  |
| Oral_1CPT_rich | 0.992 [1.0] | 3.99 [0.0] | 67 [4.0] |  |  |  |  |
| Oral_1CPT_semi_sparse | 0.968 [3.0] | 4.24 [6.0] | 68.1 [3.0] |  |  |  |  |
| Oral_1CPT_sparse1 | 2.72 [172.0] | 7.03e-73 [100.0] | 9e+27 [1.29e+28] |  |  |  |  |
| Oral_1CPT_sparse2 | 2.72 [172.0] | inf [inf] | 1.14e+22 [1.63e+22] |  |  |  |  |
| Oral_2CPT_rich | 2.72 [172.0] | 3.97 [1.0] | 111 [59.0] | 4.8e+09 [1.20e+10] | 7.25e-12 [100.0] |  |  |
|  |  |  |  |  |  |  |  |
| *Inits=nlm* |  |  |  |  |  |  |  |
| Bolus_1CPT_rich |  | 2.86 [29.0] | 2.86 [96.0] |  |  |  |  |
| Bolus_1CPT_semi_sparse |  | 2.89 [28.0] | 2.59 [96.0] |  |  |  |  |
| Bolus_1CPT_sparse1 |  | 2.29e-07 [100.0] | 0 [100.0] |  |  |  |  |
| Bolus_1CPTMM_rich |  |  | 3.38 [95.0] |  |  | 2.65 [100.0] | 2.8 [99.0] |
| Bolus_2CPT_rich |  | 2.72 [32.0] | 2.72 [96.0] | 2.72 [93.0] | 2.72 [32.0] |  |  |
| Infusion_1CPT_rich |  | 3.74 [6.0] | 90 [29.0] |  |  |  |  |
| Infusion_1CPT_semi_sparse |  | 2.81 [30.0] | 2.54 [96.0] |  |  |  |  |
| Infusion_1CPT_sparse1 |  | 3.7e-07 [100.0] | 5.13e-10 [100.0] |  |  |  |  |
| Infusion_1CPT_sparse2 |  | 6.82e-07 [100.0] | 0 [100.0] |  |  |  |  |
| Infusion_2CPT_rich |  | 2.89 [28.0] | 3 [96.0] | 2.72 [93.0] | 2.75 [31.0] |  |  |
| Oral_1CPT_semi_sparse | 2.01 [101.0] | 2.8 [30.0] | 2.55 [96.0] |  |  |  |  |
| Oral_1CPT_sparse1 | 0.0864 [91.0] | 3.33 [17.0] | 7.92 [89.0] |  |  |  |  |
| Oral_1CPT_sparse2 | 0.092 [91.0] | 3.77 [6.0] | 7.92 [89.0] |  |  |  |  |
| Oral_1CPTMM_rich | 3.01 [201.0] |  | 3.46 [95.0] |  |  | 3.37 [100.0] | 2.58 [99.0] |
| Oral_2CPT_rich | 2.72 [172.0] | 2.72 [32.0] | 2.66 [96.0] | 2.72 [93.0] | 2.72 [32.0] |  |  |
| Oral_2CPTMM_rich | 0.997 [0.0] |  | 70.5 [1.0] | 47.1 [18.0] | 4.08 [2.0] | 971 [3.0] | 240 [4.0] |
|  |  |  |  |  |  |  |  |
| *Inits=nlminb* |  |  |  |  |  |  |  |
| Bolus_1CPT_rich |  | 2.72 [32.0] | 2.72 [96.0] |  |  |  |  |
| Bolus_1CPT_semi_sparse |  | 2.2 [45.0] | 2.42 [97.0] |  |  |  |  |
| Bolus_1CPT_sparse2 |  | 5.56 [39.0] | 0.516 [99.0] |  |  |  |  |
| Bolus_1CPTMM_rich |  |  | 65.2 [7.0] |  |  | 996 [0.0] | 254 [2.0] |
| Infusion_1CPT_semi_sparse |  | 3.37 [16.0] | 2.51 [96.0] |  |  |  |  |
| Infusion_1CPTMM_rich |  |  | 45.7 [35.0] |  |  | 8e+04 [7.9e+03] | 7.1 [97.0] |
| Infusion_2CPT_rich |  | 8.91e-12 [100.0] | 79.8 [14.0] | 12.9 [68.0] | 699 [17375.0] |  |  |
| Oral_1CPT_semi_sparse | 2.27 [127.0] | 2.71 [32.0] | 2.75 [96.0] |  |  |  |  |
| Oral_1CPT_sparse1 | 2.72 [172.0] | 9.69e+15 [2.42e+17] | 54.6 [22.0] |  |  |  |  |
| Oral_1CPT_sparse2 | 0.86 [14.0] | 4.05 [1.0] | 63.5 [9.0] |  |  |  |  |
| Oral_2CPTMM_rich | 2.72 [172.0] |  | 81.5 [16.0] | 1.53e+29 [3.825e+29] | 14.9 [272.0] | 4.57 [100.0] | 2.72 [99.0] |
|  |  |  |  |  |  |  |  |
| *Inits=pipeline* |  |  |  |  |  |  |  |
| Bolus_1CPT_rich |  | 3.89 [2.7] | 66.9 [4.4] |  |  |  |  |
| Bolus_1CPT_semi_sparse |  | 4.05 [1.2] | 70.1 [0.1] |  |  |  |  |
| Bolus_1CPT_sparse1 |  | 4.1 [2.5] | 70 [0.0] |  |  |  |  |
| Bolus_1CPT_sparse2 |  | 3.95 [1.1] | 56.4 [19.4] |  |  |  |  |
| Bolus_1CPTMM_rich |  |  | 65.2 [6.9] |  |  | 998 [0.2] | 255 [1.9] |
| Bolus_2CPT_rich |  | 3.87 [3.3] | 66.2 [5.4] | 49 [22.4] | 4.05 [1.3] |  |  |
| Bolus_2CPTMM_rich |  |  | 70.9 [1.2] | 50 [24.9] | 3.94 [1.6] | 984 [1.6] | 231 [7.5] |
| Infusion_1CPT_rich |  | 4.01 [0.3] | 71.8 [2.6] |  |  |  |  |
| Infusion_1CPT_semi_sparse |  | 4.16 [4.0] | 79 [12.9] |  |  |  |  |
| Infusion_1CPT_sparse1 |  | 4.33 [8.1] | 83.5 [19.3] |  |  |  |  |
| Infusion_1CPT_sparse2 |  | 4.4 [10.0] | 78.5 [12.2] |  |  |  |  |
| Infusion_1CPTMM_rich |  |  | 70.4 [0.5] |  |  | 1.01e+03 [0.5] | 244 [2.3] |
| Infusion_2CPT_rich |  | 3.94 [1.5] | 68.2 [2.5] | 49.4 [23.6] | 3.87 [3.2] |  |  |
| Infusion_2CPTMM_rich |  |  | 70.8 [1.1] | 49.6 [23.9] | 3.89 [2.7] | 1.02e+03 [2.0] | 240 [4.1] |
| Oral_1CPT_rich | 0.996 [0.4] | 4.02 [0.4] | 66.9 [4.4] |  |  |  |  |
| Oral_1CPT_semi_sparse | 0.951 [4.9] | 4.24 [6.0] | 68.3 [2.5] |  |  |  |  |
| Oral_1CPT_sparse1 | 0.89 [11.0] | 3.92 [2.0] | 62.7 [10.4] |  |  |  |  |
| Oral_1CPT_sparse2 | 0.879 [12.1] | 4.08 [2.0] | 64.7 [7.6] |  |  |  |  |
| Oral_1CPTMM_rich | 0.974 [2.6] |  | 68.3 [2.5] |  |  | 989 [1.1] | 244 [2.4] |
| Oral_2CPT_rich | 0.93 [7.0] | 3.89 [2.8] | 68.6 [2.1] | 46.5 [16.3] | 4.27 [6.6] |  |  |
| Oral_2CPTMM_rich | 0.982 [1.8] |  | 70.9 [1.2] | 48.8 [21.9] | 3.99 [0.2] | 990 [1.0] | 251 [0.3] |

Cases those failed to get parameter estimates were not listed in the table.

^a^ "*inits = 1*" sets all initial estimates to 1, while "*inits = nls*," "*inits = nlm*," and "*inits = nlminb*" used parameter estimates from respective algorithms as initial values. "*inits = pipeline*" referred to pipeline-specific recommendations.

^b^ Values represent the estimated value, along with its relative error compared to original values in the simulated dataset. The original values using for generating simulated dataset are as follows: k_a_ = 1 h^-1^, CL = 4 L/h, V_c_ = 70 L, V_p_ = 40 L, Q = 4 L/h, V_max_ = 1000 mg/h, and K_m_ = 250 mg/L.

**Supplementary Table 5.** Statistics of final parameter estimates within 20% and 30% of original values across five initial estimate strategies in simulated datasets (SAEM)

| Dataset | K_a_ | CL | Vc | Vp | Q | V_max_ | K_m_ | V_c_ (1CPT) | V_c_ (2CPT) | All ^b^ |
| --- | --- | --- | --- | --- | --- | --- | --- | --- | --- | --- |
| Count of Estimates with Deviation ≤ 20% [% of all ] ^a^ | | | | | | | | | | |
| *Inits=1* | 3 [42.86] | 10 [66.67] | 13 [61.90] | 0 [0.00] | 5 [83.33] | 3 [50.00] | 3 [50.00] | 8 [53.33] | 5 [83.33] | 7 [33.33] |
| *Inits=nls* | 1 [14.29] | 9 [60.00] | 8 [38.10] | 0 [0.00] | 2 [33.33] | 0 [0.00] | 0 [0.00] | 6 [40.00] | 2 [33.33] | 5 [23.81] |
| *Inits=nlm* | 2 [28.57] | 10 [66.67] | 10 [47.62] | 0 [0.00] | 3 [50.00] | 3 [50.00] | 3 [50.00] | 8 [53.33] | 2 [33.33] | 7 [33.33] |
| *Inits=nlminb* | 1 [14.29] | 5 [33.33] | 6 [28.57] | 0 [0.00] | 1 [16.67] | 1 [16.67] | 1 [16.67] | 6 [40.00] | 0 [0.00] | 5 [23.81] |
| *Inits=pipeline* | **5 [71.43]]** | **15 [100.00]** | **20 [95.24]** | **1 [16.67]** | **6 [100.00]** | **6 [100.00]** | **6 [100.00]** | **14 [93.33]** | **6 [100.00]** | **13 [61.90]** |
| Count of Estimates with Deviation ≤ 30% [% of all ] | | | | | | | | | | |
| *Inits=1* | 3 [42.86] | 10 [66.67] | 14 [66.67] | 3 [50.00] | 6 [100.00] | 3 [50.00] | 3 [50.00] | 8 [53.33] | 6 [100.00] | 10 [47.62] |
| *Inits=nls* | 1 [14.29] | 9 [60.00] | 8 [38.10] | 2 [33.33] | 2 [33.33] | 0 [0.00] | 0 [0.00] | 6 [40.00] | 2 [33.33] | 7 [33.33] |
| *Inits=nlm* | 2 [28.57] | 10 [66.67] | 11 [52.38] | 2 [33.33] | 3 [50.00] | 3 [50.00] | 3 [50.00] | 8 [53.33] | 3 [50.00] | 9 [42.86] |
| *Inits=nlminb* | 1 [14.29] | 5 [33.33] | 8 [38.10] | 0 [0.00] | 1 [16.67] | 1 [16.67] | 1 [16.67] | 7 [46.67] | 1 [16.67] | 5 [23.81] |
| *Inits=pipeline* | **7 [100.00]** | **15 [100.00]** | **21 [100.00]** | **6 [100.00]** | **6 [100.00]** | **6 [100.00]** | **6 [100.00]** | **15 [100.00]** | **6 [100.00]** | **21 [100.00]** |

^a^ Count [percentage] represents the number of cases within the specified deviation threshold for each parameter and the proportion of cases where the estimates fall within the specified threshold, relative to the total number of cases where that parameter was estimated.

^b^ Values in “All” column represent the count and percentage of cases where all parameters satisfy the specified deviation threshold.

.

**Supplementary Table 6.** Statistics of final parameter estimates within 20% and 30% of original values across five initial estimate strategies in simulated datasets (FOCEI)

| Dataset | Ka | CL | Vc | Vp | Q | Vmax | Km | V_c_ (1CMPT) | V_c_ (2CMPT) | All ^b^ |
| --- | --- | --- | --- | --- | --- | --- | --- | --- | --- | --- |
| Count of Estimates with Deviation ≤ 20% [% of all ] ^a^ | | | | | | | | | | |
| *Inits=1* | 2 [28.57] | 0 [0.00] | 2 [9.52] | 0 [0.00] | 2 [33.33] | 1 [16.67] | 1 [16.67] | 0 [0.00] | 2 [33.33] | 0 [0.00] |
| *Inits=nls* | 2 [28.57] | 9 [60.00] | 8 [38.10] | 0 [0.00] | 2 [33.33] | 0 [0.00] | 0 [0.00] | 6 [40.00] | 2 [33.33] | 6 [28.57] |
| *Inits=nlm* | 1 [14.29] | 3 [20.00] | 1 [4.76] | 1 [16.67] | 1 [16.67] | 1 [16.67] | 1 [16.67] | 0 [0.00] | 1 [16.67] | 1 [4.77] |
| *Inits=nlminb* | 1 [14.29] | 2 [13.33] | 4 [19.05] | 0 [0.00] | 0 [0.00] | 1 [16.67] | 1 [16.67] | 2 [13.33] | 2 [33.33] | 2 [9.52] |
| *Inits=pipeline* | **7 [100.00]** | **15 [100.00]** | **21 [100.00]** | **1 [16.67]** | **6 [100.00]** | **6 [100.00]** | **6 [100.00]** | **15 [100.00]** | **6 [100.00]** | **16 [76.19]** |
| Count of Estimates with Deviation ≤ 30% [% of all ] | | | | | | | | | | |
| *Inits=1* | 2 [28.57] | 0 [0.00] | 2 [9.52] | 1 [16.67] | 2 [33.33] | 1 [16.67] | 1 [16.67] | 0 [0.00] | 2 [33.33] | 1 [4.76] |
| *Inits=nls* | 2 [28.57] | 9 [60.00] | 8 [38.10] | 2 [33.33] | 2 [33.33] | 0 [0.00] | 0 [0.00] | 6 [40.00] | 2 [33.33] | 8 [38.10] |
| *Inits=nlm* | 1 [14.29] | 8 [53.33] | 2 [9.52] | 1 [16.67] | 1 [16.67] | 1 [16.67] | 1 [16.67] | 1 [6.67] | 1 [16.67] | 2 [9.52] |
| *Inits=nlminb* | 1 [14.29] | 2 [13.33] | 5 [23.81] | 0 [0.00] | 0 [0.00] | 1 [16.67] | 1 [16.67] | 3 [20.00] | 2 [33.33] | 2 [9.52] |
| *Inits=pipeline* | **7 [100.00]** | **15 [100.00]** | **21 [100.00]** | **6 [100.00]** | **6 [100.00]** | **6 [100.00]** | **6 [100.00]** | **15 [100.00]** | **6 [100.00]** | **21 [100.00]** |

^a^ Count [percentage] represents the number of cases within the specified deviation threshold for each parameter and the proportion of cases where the estimates fall within the specified threshold, relative to the total number of cases where that parameter was estimated.

^b^ Values in “All” column represent the count and percentage of cases where all parameters satisfy the specified deviation threshold.

**Supplementary Table 7.** Comparison of parameter estimation results using initial estimates set to 1 vs. pipeline recommendations for one- and two-compartment models (run by SAEM)

| Dataset | *inits = 1* (1cmpt_fo) | *inits = 1* (2cmpt_fo) | *inits = pipeline* (1cmpt_fo) | *inits = pipeline* (2cmpt_fo) |
| --- | --- | --- | --- | --- |
| pheno_sd | CL = 0.00562 [2.21] ^a^ V_c_ = 1.51 [17] add = 4.29 prop = 0.165 AIC = 2959 Run time = 0.138 mins | CL = 0.00546 [2.31] V_c_ = 0.0863 [35.2] V_p_ = 1.27 [33.8] Q = 5.26 [136] add = 4.34 prop = 0.172 AIC = 6213 Run time = 0.591 mins | CL = 0.0058 [2.13] V_c_ = 1.5 [17.4] add = 4.35 prop = 0.169 AIC = 1124 Run time = 0.101 mins | CL = 0.00566 [2.69] V_c_ = 1.4 [62.8] V_p_ = 0.132 [105] Q = 0.0589 [162] add = 4.56 prop = 0.181 AIC = 1147 Run time = 0.253 mins |
| theo_sd | K_a_ = 1.42 [52.6]  CL/F = 2.8 [7.9] V_c_/F = 31.3 [1.18] add = 0.273 prop = 0.167 AIC = 368 Run time = 0.0525 mins | K_a_ = 0.166 [18.5]  CL/F = 2.77 [8.3] V_c_/F = 3.52 [30] V_p_/F = 14.2 [12.3] Q/F = 2.33 [76.5] add = 0.265 prop = 0.156 AIC = 484 Run time = 0.063 mins | K_a_ = 1.45 [50] CL/F = 2.79 [7.98] V_c_/F = 31.6 [1.18] add = 0.271 prop = 0.168 AIC = 381 Run time = 0.052 mins | K_a_ = 1.39 [70.8] CL/F = 2.81 [8.1] V_c_/F = 30.3 [4.63] V_p_/F = 0.506 [1.28e+03] Q/F = 3.61 [1.12e+03] add = 0.286 prop = 0.165 AIC = 446 Run time = 0.0861 mins |
| theo_md | K_a_ = 1.31 [55.5]  CL/F = 2.91 [6.26] V_c_/F = 31.2 [1.19] add = 0.68 prop = 0.163 AIC = 889 Run time = 0.131 mins | K_a_ = 0.158 [18.2]  CL/F = 2.89 [6.29] V_c_/F = 3.63 [28.3] Vp/F = 12.1 [15.8] Q = 1.97 [108] add = 0.641 prop = 0.159 AIC = 991 Run time = 0.155 mins | K_a_ = 1.35 [50.9] CL/F = 2.9 [6.19] V_c_/F = 31.9 [1.18] add = 0.652 prop = 0.17 AIC = 907 Run time = 0.136 mins | K_a_ = 1.24 [82.8] CL/F = 2.86 [6.66] V_c_/F = 30.2 [2.04] V_p_/F = 8.55 [130] Q/F = 0.347 [108] add = 0.685 prop = 0.161 AIC = 906 Run time = 0.199 mins |
| aprindine | K_a_ = 11 [3.97e+03]  CL/F = 102 [9.15e+03] V_c_/F = 0.0149 [6.47e+03] add = 1.75 prop = 0.00159 AIC = 1004 Run time = 0.284 mins | K_a_ = 60.9 [8.14e+03]  CL/F = 0.0184 [837] V_c_/F = 0.454 [8.62e+03] Vp/F = 424 [5.01] Q/F = 27.5 [5.7e+03] add = 0.225 prop = 0.419 AIC = 873 Run time = 0.344 mins | K_a_ = 0.369 [29.5] CL/F = 1.52 [74.6] V_c_/F = 263 [1.44] add = 0.163 prop = 0.341 AIC = 331 Run time = 0.0992 mins | K_a_ = 0.384 [34.5] CL/F = 1.22 [309] V_c_/F = 267 [2.39] V_p_/F = 98.3 [4.61e+04] Q/F = 0.0343 [1.34e+03] add = 0.168 prop = 0.318 AIC = 338 Run time = 0.164 mins |
| cefaclor | CL = 3.35e+03 [143] V_c_/F = 508 [1.36e+03] K_a_ = 0.721 [6.37e+03] add = 0.001 prop = 144 AIC = 1339 Run time = 0.284 mins | K_a_ = 253 [285]  CL/F = 9.99 [32.9] V_c_/F = 0.216 [430] Vp/F = 5.66 [89.2] Q = 7.07 [107] add = 0.001 prop = 0.497 AIC = 1083 Run time = 0.958 mins | K_a_ = 1.48 [129] CL/F = 30.9 [1.87] V_c_/F = 22.8 [14.8] add = 0.001 prop = 0.448 AIC = 692 Run time = 0.174 mins | K_a_ = 1.39 [145] CL/F = 30.7 [7.98] V_c_/F = 21.1 [16.7] V_p_/F = 24.5 [2.55e+03] Q/F = 0.245 [2.3e+03] add = 0.001 prop = 0.45 AIC = 735 Run time = 0.253 mins |
| ceftriaxone | CL/F = 225 [3.78e+03] V_c_/F = 252 [1.84e+04] add = 55.2 prop = 447 AIC = 1475 Run time = 0.0379 mins | CL/F = 290 [9.94e+06] V_c_/F = 0.553 [3.48e+07] V_p_/F = 0.364 [1.34e+07] Q/F = 0.0855 [2.46e+07] add = 51.2 prop = 3.36e+06 AIC = 11011 Run time = 0.0915 mins | CL = 0.329 [23.2] V_c_ = 1.54 [44.8] add = 42.1 prop = 0.46 AIC = 722 Run time = 0.0406 mins | CL = 0.355 [73.4] V_c_ = 0.725 [964] V_p_ = 0.712 [371] Q = 0.714 [1.55e+03] add = 44.6 prop = 0.444 AIC = 722 Run time = 0.0681 mins |
| cephalexin | K_a_ = 0.869 [51]  CL/F = 16.2 [1.36] V_c_/F = 8.16 [8.54] add = 0.001 prop = 0.425 AIC = 968 Run time = 0.153 mins | K_a_ = 814 [229]  CL/F = 7.79 [31.3] V_c_/F = 0.0134 [210] V_p_/F = 6.73 [67.5] Q/F = 11.2 [91.5] add = 0.001 prop = 0.45 AIC = 1320 Run time = 1.16 mins | Ka = 1.93 [27.7] CL/F = 16.4 [1.24] V_c_/F = 18.9 [2.88] add = 0.001 prop = 0.435 AIC = 1043 Run time = 0.166 mins | Ka = 1.12 [257] CL/F = 15.4 [1.57] V_c_/F = 11.1 [14.5] V_p_/F = 28.9 [48.2] Q/F = 2.02 [73.3] add = 0.001 prop = 0.418 AIC = 983 Run time = 0.258 mins |
| diazepam | CL = 4.2 [13.7] V_c_ = 27.7 [3.16] add = 0.0311 prop = 0.338 AIC = -256 Run time = 0.0293 mins | CL = 2.44 [17.7] V_c_ = 15.6 [6.53] V_p_ = 27.1 [7.24] Q = 10.4 [9.28] add = 0.0065 prop = 0.191 AIC = -419 Run time = 0.0439 mins | CL = 4.22 [13.7] V_c_ = 27.6 [3.17] add = 0.0313 prop = 0.337 AIC = -252 Run time = 0.0311 mins | CL = 2.42 [18.3] V_c_ = 15.7 [6.46] V_p_ = 27.4 [7.01] Q = 10.3 [9.41] add = 0.00696 prop = 0.191 AIC = -409 Run time = 0.0483 mins |
| fluorouracil | CL = 64.2 [3.17] V_c_ = 11.8 [6.58] add = 0.229 prop = 0.339 AIC = 351 Run time = 0.0293 mins | CL = 38.3 [2.59] V_c_ = 1.74 [83.5] V_p_ = 2.13 [25.5] Q = 18.3 [9.81] add = 0.269 prop = 0.302 AIC = 320 Run time = 0.0497 mins | CL = 64.3 [3.12] V_c_ = 11.7 [6.5] add = 0.244 prop = 0.342 AIC = 358 Run time = 0.0316 mins | CL = 42 [14] V_c_ = 10.6 [6.22] V_p_ = 172 [42.7] Q = 19.8 [34.3] add = 0.0291 prop = 0.373 AIC = 362 Run time = 0.0409 mins |
| oxprenolol (iv) | CL = 24.5 [1.69] V_c_ = 43.2 [0.899] add = 9.13 prop = 0.164 AIC = 1673 Run time = 0.0284 mins | CL = 12.6 [2.7] V_c_ = 1.49 [42.6] V_p_ = 13 [4.99] Q = 10 [7.67] add = 0.0232 prop = 0.389 AIC = 1712 Run time = 0.0531 mins | CL = 24.5 [1.69] V_c_ = 43.1 [0.897] add = 9.25 prop = 0.162 AIC = 980 Run time = 0.031 mins | CL = 22.9 [1.52] V_c_ = 33.6 [0.828] V_p_ = 19.5 [4.83] Q = 18.9 [6.93] add = 3.14 prop = 0.0782 AIC = 843 Run time = 0.0439 mins |
| oxprenolol (oral) | K_a_ = 0.437 [5.28]  CL/F = 58.8 [1.53] V_c_/F = 24.6 [5.2] add = 0.142 prop = 0.34 AIC = 2190 Run time = 0.0456 mins | K_a_ = 66.8 [463]  CL = 11.8 [32.8] V_c_/F = 0.0632 [433] V_p_/F = 6.54 [86.1] Q/F = 3.18 [158] add = 0.0113 prop = 0.39 AIC = 2292 Run time = 0.11 mins | Ka = 2.46 [16.2] CL/F = 58.3 [1.41] V_c_/F = 138 [1.4] add = 0.001 prop = 0.342 AIC = 2237 Run time = 0.0514 mins | Ka = 1.24 [96.1] CL/F = 49.2 [5.02] V_c_/F = 81.7 [3.59] V_p_/F = 167 [20.4] Q/F = 21.9 [10.1] add = 0.001 prop = 0.327 AIC = 2226 Run time = 0.0725 mins |
| pindolol | K_a_ = 1.32 [110]  CL/F = 24.7 [4.5] V_c_/F = 106 [2.38] add = 1.44 prop = 0.223 AIC = 651 Run time = 0.0412 mins | K_a_ = 58.6 [1.18e+03]  CL/F = 6.78 [36.5] V_c_/F = 0.203 [853] V_p_/F = 11.8 [52.2] Q/F = 2.78 [153] add = 0.775 prop = 0.339 AIC = 643 Run time = 0.0655 mins | Ka = 1.32 [107] CL/F = 24.6 [4.48] V_c_/F = 107 [2.42] add = 1.43 prop = 0.224 AIC = 668 Run time = 0.043 mins | Ka = 1.35 [90.3] CL/F = 24.6 [4.46] V_c_/F = 111 [4.26] V_p_/F = 0.251 [4.21e+03] Q/F = 12.2 [2.7e+03] add = 1.36 prop = 0.229 AIC = 676 Run time = 0.0858 mins |
| tobramycin | CL = 3.89 [4.52] V_c_/F = 25 [1.51] add = 0.001 prop = 0.259 AIC = 755 Run time = 0.344 mins | CL = 29.8 [2.8e+03] V_c_/F = 3.78 [7.98e+03] V_p_/F = 5.9 [1.1e+04] Q/F = 0.795 [8.44e+04] add = 0.001 prop = 327 AIC = 1494 Run time = 1.75 mins | CL = 3.9 [4.52] V_c_ = 25.1 [1.52] add = 0.001 prop = 0.258 AIC = 800 Run time = 0.322 mins | CL = 3.78 [4.56] V_c_ = 21.2 [1.87] V_p_ = 5.82 [12.9] Q = 0.659 [142] add = 0.001 prop = 0.248 AIC = 776 Run time = 0.669 mins |

Abbreviations^:^ 1cmpt_fo, a one-compartment model with first-order elimination (or first-order absorption and elimination in oral cases); 2cmpt_fo, a two-compartment model with first-order elimination (or first-order absorption and elimination in oral cases); Run time: computational running time; CL, clearance; V_c_, central volume of distribution; V_p_, peripheral volume of distribution; Q, inter-compartmental clearance; k_a_, absorption constant rate; add, additive residual error; prop, proportional residual error

^a.^ Parameter estimates are presented as typical population estimates with their corresponding relative standard errors (RSE%) indicated in brackets. Except for the pheno_sd case, where the unit of CL is L/h/kg and the unit of V is L/kg, the units of CL and V in all other cases are L/h and L, respectively.

**Supplementary Table 8** . Comparison between the OFV from using true values as initial estimates vs using pipeline values as initial estimate

| Dataset | OFV (from pipeline initial estimates) | OFV (from true values as initial estimates) | Difference in OFV |
| --- | --- | --- | --- |
| Bolus1CPT (rich) | 64279.84 | 64279.97 | -0.12348 |
| Bolus1CPT (semi-sparse) | 2563.402 | 2563.403 | -0.00097 |
| Bolus1CPT (sparse1) | 2309.207 | 2309.383 | -0.17631 |
| Bolus1CPT (sparse2) | 2078.655 | 2078.696 | -0.04121 |
| Bolus1CPTMM (rich) | 74197.25 | 74197.19 | 0.06 |
| Bolus2CPT (rich) | 65774.52 | 65774.55 | -0.02917 |
| Bolus2CPTMM (rich) | 70312.43 | 70312.5 | -0.06797 |
| Infusion1CPT (rich) | 62946.83 | 62946.86 | -0.02881 |
| Infusion1CPT (semi-sparse) | 2570.66 | 2570.668 | -0.00821 |
| Infusion1CPT (sparse1) | 2311.72 | 2311.654 | 0.066776 |
| Infusion1CPT (sparse2) | 2096.266 | 2096.064 | 0.201977 |
| Infusion1CPTMM (rich) | 78911.61 | 78911.7 | -0.08985 |
| Infusion2CPT (rich) | 64981.12 | 64981.22 | -0.10016 |
| Infusion2CPTMM (rich) | 75074.48 | 75074.66 | -0.17993 |
| Oral1CPT (rich) | 62205.02 | 62205.12 | -0.10444 |
| Oral1CPT (semi-sparse) | 2549.289 | 2549.294 | -0.00564 |
| Oral1CPT (sparse1) | 3592.911 | 3592.901 | 0.010571 |
| Oral1CPT (sparse2) | 3318.832 | 3318.796 | 0.035833 |
| Oral1CPTMM (rich) | 72361.53 | 72361.51 | 0.022565 |
| Oral2CPT (rich) | 64213.21 | 64213.29 | -0.08108 |
| Oral2CPTMM (rich) | 69533.43 | 69533.46 | -0.03355 |

OFV: objective function values. Difference in OFV: OFV (from pipeline) – OFV (from true values). Re-estimation was run by FOCEI

**Supplementary Table 9.** Initial estimates recommended by the pipeline for 13 real-life datasets

| Datasets | Methods for 1CMPT parameters | k_a_ | CL | V_c_ (1CMPT) | V_c_ (2CMPT) | V_p_ | Q |
| --- | --- | --- | --- | --- | --- | --- | --- |
| pheno_sd | Adaptive single-point methods |  | 0.0087 | 1.25 | 1.25 | 0.125 | 0.0174 |
| theo_sd | Naive pooled NCA (FD) | 1.58 | 3.04 | 33 | 31.9 | 3.19 | 6.08 |
| theo_md | Adaptive single-point methods | 1.35 | 2.37 | 33.5 | 33.5 | 3.35 | 2.38 |
| aprindine | Adaptive single-point methods | 0.551 | 3.45 | 337 | 337 | 33.7 | 1.185 |
| cefaclor | Hybrid methods | 2.06 | 29.7 | 33.3 | 33.3 | 16.65 | 14.85 |
| ceftriaxone | Naive pooled NCA (FD) |  | 0.164 | 1.68 | 1.68 | 0.336 | 0.164 |
| cephalexin | Hybrid methods | 1.14 | 19.5 | 18.3 | 11.2 | 5.6 | 19.5 |
| diazepam | Naive pooled NCA (FD) |  | 2.51 | 58.5 | 19 | 19.0 | 5.02 |
| fluorouracil | Naive pooled NCA (FD) |  | 70.3 | 14 | 21.6 | 216 | 35.15 |
| oxprenolol (iv) | Naive pooled NCA (FD) |  | 21.9 | 61.4 | 37.8 | 18.9 | 10.95 |
| oxprenolol (oral) | Graphic methods | 1.48 | 58.2 | 169 | 169 | 338 | 29.1 |
| pindolol | Graphic methods | 2.47 | 24.8 | 148 | 145 | 14.5 | 49.6 |
| tobramycin | Naive pooled NCA (MD) |  | 3.95 | 29.5 | 29.5 | 2.95 | 1.975 |

1CMPT: one-compartment model; 2CMPT: two-compartment model

Hybrid methods: parameter combinations were drawn from multiple candidate methods, rather than a single unified method.

Except for the pheno_sd case, where the unit of CL is L/h/kg and the unit of V is L/kg, the units of CL and V in all other cases are L/h and L, respectively.

**Reference**

1. Hagemeijer F (1975) Absorption, half-life, and toxicity of oral aprindine in patients with acute myocardial infarction. Eur J Clin Pharmacol 9:21–25. https://doi.org/10.1007/BF00613425

2. Korzeniowski OM, Scheld WM, Sande MA (1977) Comparative Pharmacology of Cefaclor and Cephalexin. Antimicrob Agents Chemother 12:157–162. https://doi.org/10.1128/aac.12.2.157

3. Martin E, Koup JR, Paravicini U, Stoeckel K (1984) Pharmacokinetics of ceftriaxone in neonates and infants with meningitis. J Pediatr 105:475–481. https://doi.org/10.1016/S0022-3476(84)80032-3

4. Ghoneim MM, Mewaldt SP, Ambre J (1975) Plasma Levels of Diazepam and Mood Ratings. Anesth Analg 54:173

5. Kaplan SA, Jack ML, Alexander K, Weinfeld RE (1973) Pharmacokinetic profile of diazepam in man following single intravenous and oral and chronic oral administrations. J Pharm Sci 62:1789–1796. https://doi.org/10.1002/jps.2600621111

6. MacMillan WE, Wolberg WH, Welling PG (1978) Pharmacokinetics of Fluorouracil in Humans1. Cancer Res 38:3479–3482

7. Phillips TA, Howell A, Grieve RJ, Welling PG (1980) Pharmacokinetics of oral and intravenous fluorouracil in humans. J Pharm Sci 69:1428–1431. https://doi.org/10.1002/jps.2600691220

8. Mason WD, Winer N (1976) Pharmacokinetics of oxprenolol in normal subjects. Clin Pharmacol Ther 20:401–412. https://doi.org/10.1002/cpt1976204401

9. Gugler R, Herold W, Dengler HJ (1974) Pharmacokinetics of pindolol in man. Eur J Clin Pharmacol 7:17–24. https://doi.org/10.1007/BF00614385

10. Aarons L, Vozeh S, Wenk M, et al (1989) Population pharmacokinetics of tobramycin. Br J Clin Pharmacol 28:305–314. https://doi.org/10.1111/j.1365-2125.1989.tb05431.x
